# Differential regulation of degradation and immune pathways underlies adaptation of the ectosymbiotic nematode *Laxus oneistus* to oxic-anoxic interfaces

**DOI:** 10.1038/s41598-022-13235-9

**Published:** 2022-06-13

**Authors:** Gabriela F. Paredes, Tobias Viehboeck, Stephanie Markert, Michaela A. Mausz, Yui Sato, Manuel Liebeke, Lena König, Silvia Bulgheresi

**Affiliations:** 1 Department of Functional and Evolutionary Ecology, Environmental Cell Biology Group, University of Vienna, Vienna, Austria; 2 Vienna Doctoral School of Ecology and Evolution, Vienna, Austria; 3 Division of Microbial Ecology, Center for Microbiology and Environmental Systems Science, University of Vienna, Vienna, Austria; 4 Department of Pharmaceutical Biotechnology, Institute of Pharmacy, University of Greifswald, Greifswald, Germany; 5 School of Life Sciences, University of Warwick, Coventry, UK; 6 Max Planck Institute for Marine Microbiology, Bremen, Germany

**Keywords:** Metabolism, Antimicrobial responses, Bacteria

## Abstract

Eukaryotes may experience oxygen deprivation under both physiological and pathological conditions. Because oxygen shortage leads to a reduction in cellular energy production, all eukaryotes studied so far conserve energy by suppressing their metabolism. However, the molecular physiology of animals that naturally and repeatedly experience anoxia is underexplored. One such animal is the marine nematode *Laxus oneistus*. It thrives, invariably coated by its sulfur-oxidizing symbiont *Candidatus* Thiosymbion oneisti, in anoxic sulfidic or hypoxic sand. Here, transcriptomics and proteomics showed that, whether in anoxia or not, *L. oneistus* mostly expressed genes involved in ubiquitination, energy generation, oxidative stress response, immune response, development, and translation. Importantly, ubiquitination genes were also highly expressed when the nematode was subjected to anoxic sulfidic conditions, together with genes involved in autophagy, detoxification and ribosome biogenesis. We hypothesize that these degradation pathways were induced to recycle damaged cellular components (mitochondria) and misfolded proteins into nutrients. Remarkably, when *L. oneistus* was subjected to anoxic sulfidic conditions, lectin and mucin genes were also upregulated, potentially to promote the attachment of its thiotrophic symbiont. Furthermore, the nematode appeared to survive oxygen deprivation by using an alternative electron carrier (rhodoquinone) and acceptor (fumarate), to rewire the electron transfer chain. On the other hand, under hypoxia, genes involved in costly processes (e.g., amino acid biosynthesis, development, feeding, mating) were upregulated, together with the worm’s Toll-like innate immunity pathway and several immune effectors (e.g., bactericidal/permeability-increasing proteins, fungicides). In conclusion, we hypothesize that, in anoxic sulfidic sand, *L. oneistus* upregulates degradation processes, rewires the oxidative phosphorylation and reinforces its coat of bacterial sulfur-oxidizers. In upper sand layers, instead, it appears to produce broad-range antimicrobials and to exploit oxygen for biosynthesis and development.

## Introduction

Fluctuations that lead to a decrease in oxygen availability are common in nature^1^. The physiological and behavioral response to oxygen deprivation has been studied in animals that naturally experience oxygen deprivation, such as frogs, goldfish, and turtles^1–3^, as well as in invertebrate genetic models^4–6^. When oxygen deprived, these organisms must face the challenge of a drastic drop in the production of the energy-storing metabolite adenosine triphosphate (ATP), which leads to the failure of energy-demanding processes that are crucial for maintaining cellular homeostasis. Anoxia-tolerant organisms, however, are capable of saving energy by stopping energy-costly cellular functions (e.g., protein synthesis, ion pumping, and cell cycle progression), maintaining stable and low permeability of membranes, and producing ATP by anaerobic glycolysis^2,6,7^.

When parasitic and free-living nematodes, including the model organism *Caenorhabditis elegans*, are experimentally exposed to anoxia (< 0.001 kPa O_2_), the intracellular ATP/ADP ratio drops dramatically and, within 10 h, they enter a state of reversible metabolic arrest called *suspended animation*. Once in this state, *C. elegans* stop to eat, move, develop or lay eggs, implying that oxygen deprivation affects their growth and behavior^8–10^. If these effects can be reversed upon oxygen reestablishment, the latter can also provoke a massive and sudden production of reactive oxygen species (ROS) that may overwhelm the organism’s antioxidant defense, and cause its death (reviewed in^1^). Of note, an increase in mitochondrial ROS production was also observed in worms under hypoxia, because of the inefficient transfer of electrons to molecular oxygen^11,12^.

Because oxygen diffuses slowly through aqueous solutions, sharp concentration gradients of this electron acceptor may occur in marine environments and wet soil^13^. It is at oxic-anoxic interfaces of marine sands that free-living nematodes coated with sulfur-oxidizing Gammaproteobacteria (Stilbonematinae) abound^14–17^. However, up to this study, the molecular mechanisms allowing symbiotic nematodes to withstand anoxia, and the inherent stress it is known to inflict upon metazoans, were unknown. Here, we incubated *Laxus oneistus*^18^ in conditions resembling those it encounters in its natural environment (i.e., anoxic sulfidic or hypoxic), and applied comparative transcriptomics, proteomics and lipidomics to understand how it copes with oxygen deprivation. Contrarily to our expectations, in anoxic sulfidic water *Laxus oneistus* did not enter suspended animation. However, it upregulated genes required for ribosome biogenesis and energy generation, and for degradation pathways (e.g., ubiquitination-proteasome systems, autophagy) likely involved in recycling damaged cellular components and misfolded proteins into nutrients. Notably, under anoxic sulfidic conditions, it also upregulated putative symbiont-binding molecules such as lectins. In the presence of oxygen, on the other hand, the worm appeared to overexpress genes involved in energy-demanding processes (e.g., amino acid synthesis, development, feeding, and mating) and upregulated the synthesis of broad-range antimicrobials, likely via triggering the Toll/NF-kB pathway.

## Results and discussion

### The nematode *Laxus oneistus* did not enter suspended animation upon 24 h in anoxia

To survive anoxia, nematodes enter suspended animation to suppress metabolism and conserve energy. The most notorious sign of suspended animation is the arrest of motility^5,10^.

Surprisingly, although the whole population of four tested nematode species, including *C. elegans*, was reported to be in suspended animation upon 10 h in anoxia^10^, *L. oneistus* kept moving not only after 24-h-long incubations, but also upon 6-day-long incubations in anoxic seawater (three batches of 50 worms were incubated under each condition). Additionally, the symbiotic nematodes appeared morphologically normal (Supplemental Movies 1–4).

The fact that we could not observe suspended animation, led us to hypothesize that *L. oneistus* evolved unprecedented strategies to survive oxygen deprivation.

### Stable transcriptional profile under hypoxic or anoxic sulfidic conditions

To understand the molecular mechanisms underlying *L. oneistus* response to oxygen, we subjected it to various oxygen concentrations. Namely, nematode batches were incubated under either normoxic (100% air saturation; O), hypoxic (30% air saturation; H) or anoxic (0% air saturation; A) conditions for 24 h. Additionally, given that *L. oneistus* thrives in reduced sand containing up to 25 µM sulfide^16,17^, we also incubated it in anoxic seawater supplemented with < 25 µM sulfide (anoxic sulfidic condition; AS; see^16^ for the sulfide concentration experienced by *L. oneistus* during each incubation).

Whereas transcriptional differences of the symbiont *Candidatus* Thiosymbion oneisti incubated under normoxic (O) and hypoxic (H) conditions were negligible^16^, the expression profiles of nematode batches incubated under O conditions varied so much that they did not cluster (Fig. S1). Consequently, there was no detectable differential expression between the transcriptomes of O nematodes and any of the other transcriptomes (H, A or AS; Fig. S1B, C). We attribute the erratic transcriptional response of *L. oneistus* to normoxia to the fact that this concentration is not naturally experienced by *L. oneistus*^16,17^.

As for the expression profiles of nematodes subjected to the H, A or AS conditions, replicates of each condition behaved more congruently (Fig. S1B). However, we did not find any significant difference between the A and AS nematodes and only 0.05% of the genes (8 genes; Data S1) were differentially expressed between the H and A nematodes. Moreover, even at the proteome level, there was no significant difference between the H and A incubations (t-test, Benjamini–Hochberg correction, p < 0.05; Fig. S2A, Data S1). Finally, only the comparison of the AS and H transcriptomes resulted in 4.8% of the expressed genes (787 out of 16,526) being differentially expressed, with 434 genes being upregulated under the AS condition and 353 being upregulated under the H condition (Fig. S1C, Data S1).

Collectively, our data suggests that *L. oneistus* may be ill-equipped to handle normoxic sediment, but it maintains a largely stable physiological profile under all other conditions. Before discussing the subset of biological processes differentially upregulated in AS versus H nematodes and vice versa, we will present the physiological processes the worm appears to mostly engage with, irrespective of the environmental conditions we experimentally subjected it to.

### Top-expressed transcripts under all tested conditions

**Figure 1:**
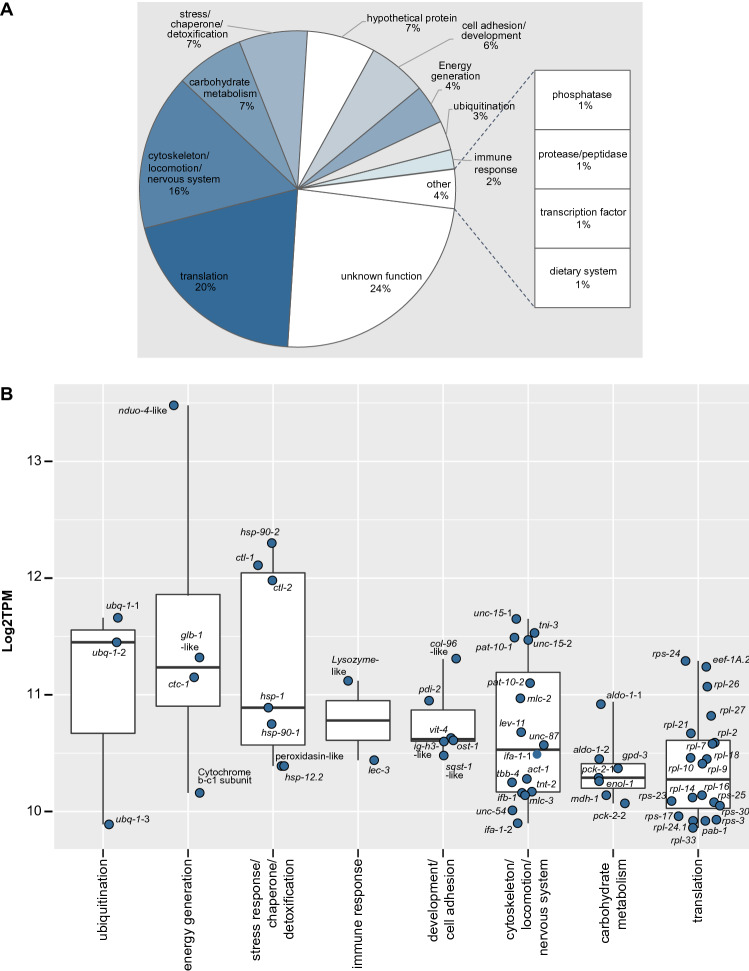
Relative transcript abundance and expression levels of the top 100 expressed genes of *L. oneistus* across all conditions.** (A**) Relative transcript abundance (%) of the top 100 expressed genes with a manually curated functional category. The top 100 expressed genes were collected by averaging the expression values (log_2_TPM) across all replicates of all incubations (Fig. S1A, Data S1, and S2). Functional classifications were extracted from UniProt and from comprehensive literature search focused mainly on *C. elegans,* and confirmed with the automatically annotated eggNOG classification (Data S1). (**B**) Median gene expression levels of selected *L. oneistus* manually annotated functional categories of the top 100 expressed genes. Metabolic processes include both differentially and constitutively expressed genes. Each dot represents the average log_2_TPM value per gene across all replicates of all incubations. All gene names (or locus tags for unidentified gene names) are listed in Data S2.

To gain insights on *L. oneistus* basal physiology, we treated all 16 transcriptomes as biological replicates (i.e., O, H, A and AS transcriptomes were pooled) and identified the 100 most abundant transcripts out of 16,526 based on functional categories extracted from the UniProt database^19^ and comprehensive literature search (Fig. 1, Data S2). Our manual classification was supported by automatic eggNOG classification (Data S2). Similarly, the H and A proteomes were pooled, and the 100 most abundant proteins out of 2,626 were detected (Fig. S2).

Based on median gene expression values of the top 100 expressed genes, we found that some of the processes *L. oneistus* mostly engages with were ubiquitination (*ubq-1*^20^), energy generation (e.g., globin *glb-1*-like^21^), cytochrome c oxidase I subunit *ctc-1* (UniProtKB P24893), *nduo-4*-like (UniProtKB P24892), stress response and detoxification (e.g., *hsp-1*, *hsp-90*, *hsp12.2*, and catalases *ctl-1* and *ctl-2*^22,23^), and immune defense (lysozyme-like proteins and *lec-3*) (Fig. 1, Data S2).

Lastly, 48 out of the top 100 most expressed genes, were also detected among the top 100 proteins (Fig. 1, Fig. S2, and Data S2, Supplemental material). Despite the modest Spearman correlation between transcript and protein levels (ρ = 0.4, *p*-value < 0.01) (Fig. S3A), there was an overlap in the detected biological processes (e.g., energy generation, stress response or detoxification categories, carbohydrate metabolism, cytoskeleton, locomotion, nervous system) (Figs. S2, S3B). All in all, except for those encoding for immune effectors, top-transcribed *L. oneistus* genes could not be unambiguously ascribed to its symbiotic lifestyle. This differs to what has been observed for other chemosynthetic hosts, such as giant tubeworms and clams. It is perhaps because these animals acquire their endosymbionts horizontally and feed on them (as they are housed intracellularly) that they abundantly express genes involved in symbiont acquisition, proliferation control and digestion^24–26^. Notably, we did observe a partial overlap of the most expressed gene categories (e.g., oxidative stress, energy generation, immune response), when *L. oneistus* was compared to the marine gutless annelid *Olavius* a*lgarvensis*. We ascribe the overlap to the fact that, albeit endosymbiotic, *O.* a*lgarvensis* also inhabits shallow water sand (Fig. S4, Supplemental material) and, as hypothesized for *L. oneistus*, it may also acquire its symbionts vertically^27–29^.

**Figure 2:**
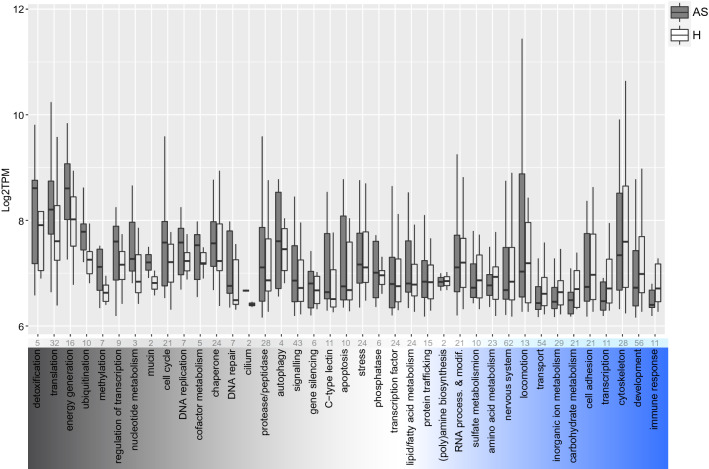
Median gene expression levels of selected *L. oneistus* metabolic processes among the differentially expressed genes between the hypoxic (H) and anoxic sulfidic (AS) conditions after 24 h. Individual processes among the differentially expressed genes are ordered according to their difference in median expression between the AS and H incubations. Namely, detoxification (far left) had the largest difference in median expression in the AS condition, whereas immune response (far right) had the largest median expression difference in the H condition. The absolute number of genes are indicated at the top of each process. Metabolic processes were manually assigned and confirmed with the automatic annotated eggNOG classification. For specific gene assignments see Data S1. Some genes are present in more than one functional category and processes comprising only one gene are not displayed in the figure but listed in Data S1.

To conclude, although both symbiont^16^ and host transcriptomics do not suggest a high degree of inter-partner metabolic dependence in the *L. oneistus* ectosymbiosis, the nematode seems well-adapted to both anoxic sulfidic (AS) and hypoxic (H) sand (Fig. 2, Data S1). The transcriptional response of the worm to these two conditions is, however, significant (Fig. 2, Data S1), and it will be reported below.

### Genes upregulated in anoxic sulfidic (AS) nematodes

#### Chaperones and detoxification

**Figure 3:**
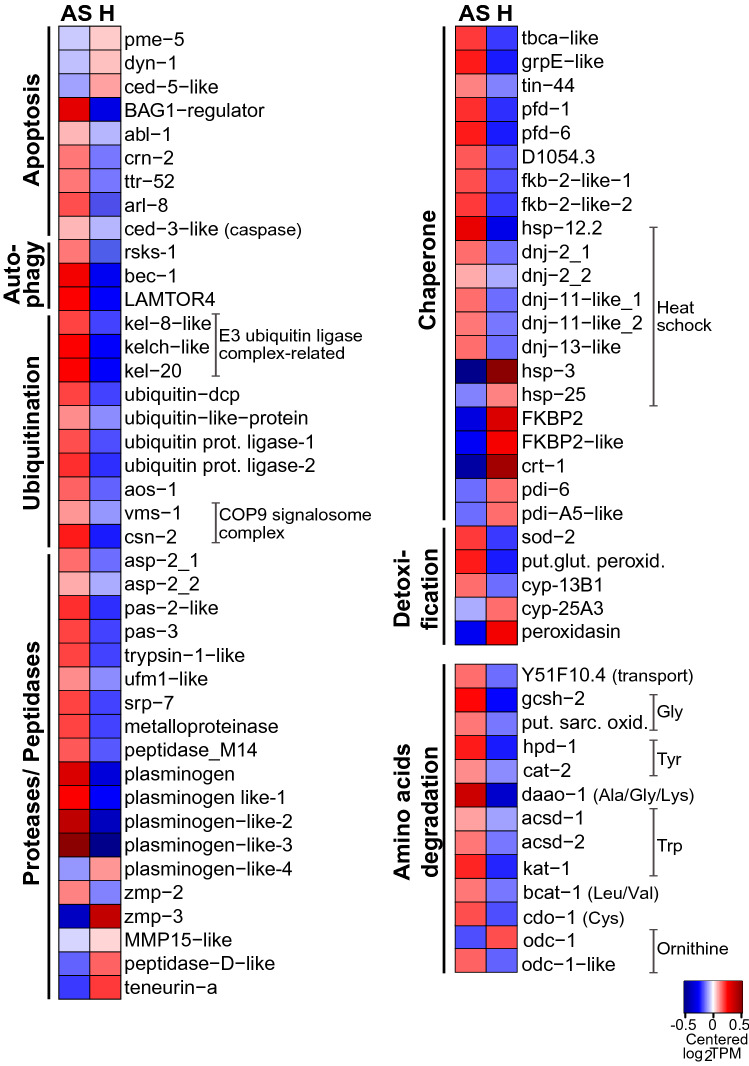
Genes involved in detoxification, ubiquitin–proteasome, autophagy, apoptosis, and amino acids degradation were predominantly expressed in AS worms. Heatmap displaying genes upregulated in AS (anoxic sulfidic) relative to H (hypoxic) worms after 24 h-long incubations under one of the two conditions (1.5-fold change, FDR ≤ 0.05). Expression levels are displayed as mean-centered log_2_TPM value (transcripts per kilobase million). Genes are ordered by function in their respective metabolic pathways. For each process, the minority of genes that were upregulated in H worms is shown in Data S1. Red denotes upregulation, and blue downregulation. Prot. protein, COP9: Constitutive photomorphogenesis 9. dcp: domain-containing proteins; Put. glut. peroxid.: putative glutamate peroxidase; Put. sarc. oxid.: putative sarcosine oxidase.

The expression of chaperone-encoding (e.g., *hsp12.2*, *grpE*, *dnaJ/dnj-2*, *pfd-1*, *pfd-6*^30–32^) and ROS-detoxification-related genes (e.g., superoxide dismutase *sod-2* and a putative glutathione peroxidase, involved in the detoxification of superoxide byproducts and hydrogen peroxide, respectively^33^) were higher in AS nematodes (Figs. 2 and 3). Notably, transcripts encoding for the heme-binding cytochrome P450 *cyp*-13B1 were also more abundant in AS (Fig. 3), perhaps to increase the worm’s capacity to cope with putative ROS formation^34^. Indeed, as cells start being oxygen-depleted, mitochondrial ROS accumulate because of the inefficient transfer of electrons to molecular oxygen^11,12^. Alternatively, the upregulation of antioxidant-related genes in AS worms could represent an anticipation response to an imminent reoxygenation. In animals alternating between anoxic and oxygenated habitats, the re-exposure to oxygen can be very dangerous, as it creates a sudden ROS overproduction that may overwhelm the oxidative defense mechanisms^1^. Although it has not been reported for nematodes, overexpression of ROS-counteracting genes is consistent with what has been reported for vertebrates and marine gastropods which, just like *L. oneistus*, alternate between oxygen-depletion and reoxygenation^1^.

#### Mitochondrial and cytoplasmic ribosome biogenesis

**Figure 4:**
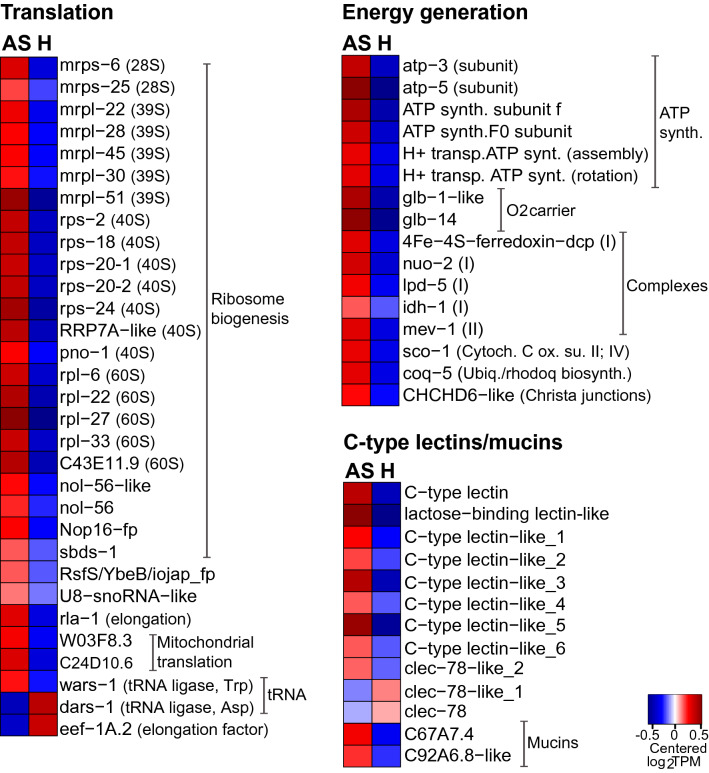
Genes involved in translation and energy generation and genes encoding for C-type lectins and mucins were predominantly expressed in AS worms. Heatmap displaying genes upregulated in AS (anoxic sulfidic) relative to H (hypoxic) worms, upon 24 h-long incubations under one of the two conditions (1.5-fold change, FDR ≤ 0.05). Expression levels are displayed as mean-centered log_2_TPM values (transcripts per kilobase million). Genes are ordered by function in their respective metabolic pathways. For each process, the minority of genes that were upregulated in H worms is shown in Data S1. Red denotes upregulation, and blue downregulation. Fp, family-containing protein; Cytoch. C ox. su. II: cytochrome c oxidase subunit II; Ubiq./rhodoq biosynth.: Ubiquinone or rhodoquinone biosynthesis.

In the cellular stress imposed by oxygen deprivation, mitochondria are central to both death and survival (reviewed in^7^). In this scenario, calcium regulation, the scavenging of ROS or the suppression of their production, and/or inhibition of the mitochondrial permeability transition pore (MPTP) opening, might help to preserve mitochondrial function and integrity^7,35^. In addition, removal of specific mitochondrial components (mitochondrial-associated protein degradation, MAD), might also arise to maintain the overall mitochondrial homeostasis^36^. Perhaps as a response to anoxia-induced stress (reviewed in^7^), a gene involved in MAD (*vms-*1)^36^ was upregulated in AS worms (Fig. 4). More abundant in this condition were also transcripts encoding for mitochondrial transmembrane transporters *tin-44*, *slc-25A26* and *C16C10.1* (UniProtKB O02161, Q18934, Q09461), putatively transporting peptide-containing proteins from the inner membrane into the mitochondrial matrix, such as S-Adenosyl methionine (Fig. 6). Surprisingly, although the translation elongation factor *eef-1A.2*^37^ was downregulated in AS worms, not only various mitochondrial ribosome structural components (28S: *mrps*, 39S: *mrpl*)^38^, and mitochondrial translation-related genes (e.g., *C24D10.6* and *W03F8.3)*^39^ were upregulated in AS nematodes, but also several cytoplasmic ribosome biogenesis (40S: *rps*, 60S: *rpl*)^40^ and subunit assembly genes (e.g., RRP7A−like)^41^ (Fig. 4).

Taken together, the maintenance of mitochondrial homeostasis, an anticipatory response to a potential upcoming ROS insult (see “Chaperones and detoxification” section) and/or their involvement in extra-ribosomal functions^42,43^ might explain the upregulation of ribosomal biogenesis-related genes in AS nematodes. Although upregulation of ribosomal proteins has also been observed in gastropods exposed to anoxia^44^, increased ribosomal biogenesis (which oftentimes directly correlates with an increase in protein synthesis) is not expected in animals that must repress their metabolism to cope with oxygen deprivation^3^.

#### Energy generation

Equally surprising was the upregulation of all differentially expressed genes related to energy generation in AS nematodes (Fig. 4). Namely, besides putative oxygen-binding globulin-like genes (e.g., *glb-1*, *glb-14*)^21^, the following were upregulated in AS nematodes: key structural genes (e.g., *atp-3*, *atp-5*)^45^, assembly-related genes (H^+^-transport ATP synthase)^46^ of the mitochondrial ATP synthase (complex V), genes related to complex I (*lpd-5*, *nuo-2*)^47,48^, a subunit of the succinate dehydrogenase involved in complex II (*mev-1*)^49^, a mitochondrial cytochrome c oxidase subunit II assembly gene related to complex IV (*sco-1*)^50^, and a mitochondrial gene (*coq-5*), involved in the synthesis of either ubiquinone (Q, aerobic) or rhodoquinone (RQ, anaerobic) electron carriers^51^ (Fig. 4). This suggests that, under anoxia, the electron transfer chain (ETC) is rewired: the electrons still enter the ETC at complex I, but do not reach complex III and IV. Instead, complex II, which in aerobic conditions functions as a succinate dehydrogenase, is repurposed into a reductase, which shuttles electron from the electron donor rhodoquinone to the electron acceptor fumarate. This mechanism would maintain the flow of electrons through the ETC, and it would prevent mitochondrial ATP generation (complex V) from shutting down^51,52^.

In short, under AS, similarly to what has been observed in other free-living and parasitic nematodes, complex I appears to be the sole proton pump in this truncated form of ETC^51,52^. In accordance with this hypothesis, tryptophan (Trp) degradation-related genes (*acsd-1*, *acsd-2*) and the Trp RNA ligase (*wars-1*)^53^ that might be required to synthesize RQ^51,52^ were upregulated under AS. Intriguingly, an isocitrate dehydrogenase gene (*idh-1*) was also upregulated. This produces reducing equivalent (NADPH) carrying electrons that may fuel complex I^54^, but it might also add to the stimulation of the antioxidant capacity or to the maintenance of redox homeostasis by regenerating reduced glutathione^1,55^.

If glycolysis is a key process for ATP generation in anoxia^3^ and if, consistently, *hxk-2* was upregulated under this condition (Fig. 6), based on the expression levels of transcripts encoding for alpha-amylases (see “Carbohydrate metabolism” in Fig. 6), starch and/or glycogen^56^ may be the prominent carbon sources under anoxic sulfidic conditions.

#### Ubiquitin–proteasome system and proteases

Proteolysis supplies amino acids or polypeptides to the cells, while impeding the accumulation of damaged or misfolded proteins. The two main mechanisms of cellular proteolysis are the lysosome-mediated intracellular protein degradation (autophagy) and the proteasome-mediated protein degradation (ubiquitin–proteasome system, UPS). In the latter, ubiquitin-protein ligases covalently attach ubiquitin to proteins, allowing their recognition and further degradation by the proteasome^57^.

As shown in Fig. 1, transcripts encoding for polyubiquitin (*ubq-1*), had the highest median gene expression across all transcriptomes. However, all ubiquitination-related genes detected in the differential gene expression analysis between the AS and H conditions, were upregulated in AS worms (Fig. 2 and 3, Data S1). For example, *aos-1,* encoding for a subunit of the ubiquitin-activating enzyme (E1)^58^, two ubiquitin-protein ligases (E3s without detected cullin domains)^57^, and kelch-like genes (e.g., *kel-8*-like and *kel-20*). The former are BTB-domain containing proteins known to interact with E3 enzymes, with *kel-8* being involved in the degradation of glutamate neuroreceptors^59^. Additional ubiquitination-related genes upregulated in AS were *csn-2*, encoding for a component of the COP9 signalosome complex^60^, and proteasome genes (*pas-2* and *pas-3*)^57^.

Among the proteases that were upregulated in AS worms, aspartyl proteases have been involved in neurodegeneration^61^, whereas plasminogen and the zinc matrix metalloproteinase ZMP-2 were both reported to mediate degradation of extracellular matrix (ECM)^62^ (Fig. 3). *C. elegans* ZMP-2 was also shown to prevent the accumulation of oxidized lipoproteins^62^, and therefore, it may contribute to the enhanced antioxidant response observed in this condition.

#### Autophagy and amino acid degradation

Besides acting coordinately to withstand stress, autophagy cooperates with apoptotic UPS for the recovery and supply of nutrients when these are scarce (reviewed in^63,64^). Transcripts of two autophagy-related genes, *bec-1*^65^ and the Ragulator complex protein LAMTOR4 (C7orf59-like)^66^ were more abundant in AS nematodes (Fig. 3). While the former positively regulates autophagy^65^, the latter interacts with the mTOR Complex I (mTORC1), and tethers small GTPases (Rags and Rheb) to the lysosomal surface^66^. When amino acid levels are low, mTORC1 is not translocated to the lysosomal surface^66^, thereby favoring catabolic processes such as autophagy^67^. We propose that amino acid scarcity might result from the upregulation of genes involved in the degradation of lysin, glycin, tyrosin, cystein, leucin, isoleucin, valin or tryptophan (Fig. 3, Data S1). This would decrease mTORC1 activity and, in turn, stimulates nutrient recycling via autophagy in AS worms.

Conversely, we hypothesize that in H worms, active mTORC1 interacts with the ribosomal protein S6 kinase (S6K), encoded by the *rsks-1* gene which is also up in H worms^68^ (Fig. 3). This direct interaction, upon a cascade of phosphorylation events, would stimulate translation, and ultimately cell growth and proliferation^68,69^.

All in all, although it is currently unclear whether increased autophagy is beneficial or detrimental, under AS conditions, the upregulation of genes involved in self-digestion might play a protective role and foster recovery from starvation^67^, pathogens^70^ or from neuronal and muscular degeneration induced by oxygen deprivation^35^.

#### Lectins and mucins

Given that symbiont attachment may be mediated by Ca^2+^-dependent lectins^71–73^ and that, under anoxia, the symbiont appeared to proliferate more^16^, we expected nematode C-type lectins to be upregulated under this condition. Indeed, nine C-type lectin domain (CTLD)-containing proteins were upregulated in AS *L. oneistus* adults and only two (*clec-78* and *clec-78*-like-2) were upregulated in the presence of oxygen (Fig. 4). In addition to CTLD-containing proteins, mucins, a class of glycoproteins with more than 50% of its mass attributable to O-glycans, were also upregulated in AS nematodes. Considering that mucin glycans are used by vertebrate gut commensals for attachment, as well as being a source of nutrients^74^, it is conceivable that their upregulation in anoxia (Fig. 4), together with that of CTLD-containing proteins, would foster symbiont attachment.

We hypothesize that overexpression of two classes of putative symbiont-binding molecules, lectins and mucins, under conditions favoring symbiont proliferation (i.e., AS conditions)^16^, may mediate bacterial coat reinforcement.

#### Apoptosis

Mitochondria play an important role in apoptosis induction^54^. Indeed, MPTP opening due to ROS (or the severe ATP decline imposed by the absence of oxygen) may cause cytochrome c release from mitochondria and this, in turn, triggers caspase activation^7^. We observed that transcripts encoding for *sco-1*, a gene needed for the synthesis and assembly of mitochondrial cytochrome c^50^, were more abundant in AS worms (Fig. 4). Further, we observed upregulation of Caspase-3 (*ced-3*) which belongs to a family of cysteine proteases involved in apoptosis and which is activated upon mitochondrial cytochrome c release into the cytosol^54,75^. Additional apoptosis-related genes that appeared to be upregulated in AS worms were: *bec-1* (Fig. 3), a gene that promotes autophagy and fine-tunes the Ced-3-mediated apoptosis^65^; *ttr-52*, which mediates apoptotic cell recognition prior to engulfment^76^; a BAG family molecular chaperone regulator 1 (BAG1-regulator); a celldeath-related nuclease *crn−2*^77^ and phagolysosome forming *arl-8*^78^, and a tyrosine-protein kinase (*abl-1*) that modulates apoptotic engulfment pathways^79^.

#### Lipid catabolism

Genes involved in lipid metabolism were similarly expressed between the AS and H conditions (Fig. 2, Data S1). In accordance, lipidomes of nematodes incubated in the presence or absence of oxygen were not significantly different (Fig. S5, Supplemental material). However, in line with the overall upregulation of degradation pathways, we observed upregulation of genes involved in fatty acid beta-oxidation (*kat-1*)^80^, in lipid digestion (the lipase *lipl-6*; UniProtKB E2S7J2), and lipid degradation (a peripilin-2-like protein)^81^. Moreover, a gene that might be involved in oxidative stress tolerance (a stearic acid desaturase *fat-7* regulating the first step of the fatty acid desaturation pathway^82^ was also upregulated in AS worms. Lipid degradation under anoxia might be a strategy to overcome starvation^83^.

**Figure 5:**
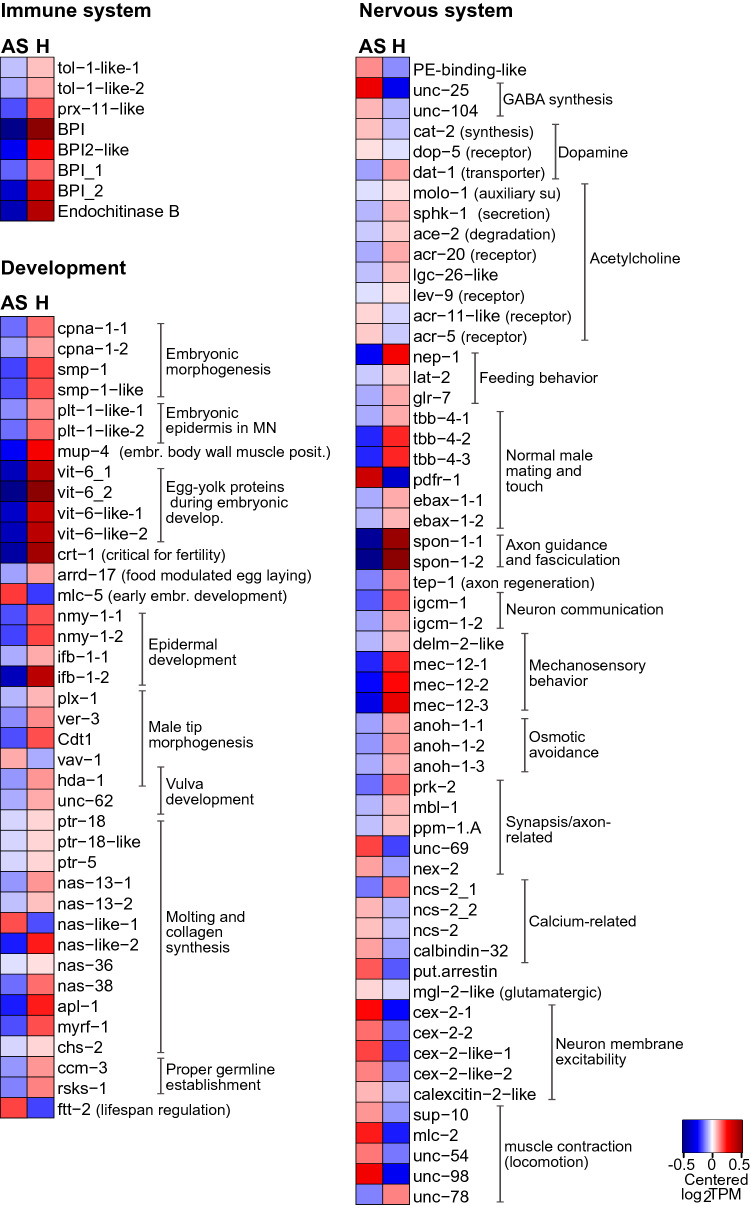
Genes involved in immune response, development and nervous system were predominantly expressed in hypoxic (H) worms. Heatmap displaying genes upregulated in H relative to AS worms, upon 24 h-long incubations under one of the two conditions (1.5-fold change, FDR ≤ 0.05). Expression levels are displayed as mean-centered log_2_TPM value (transcripts per kilobase million). Genes are ordered by function in their respective metabolic pathways. For each process, the minority of genes that were upregulated in AS worms is shown in Data S1. Red denotes upregulation and blue downregulation. MN, mechanosensory neurons; Embr. body wall muscle posit.: Embryonic body wall muscle positioning; Put.; putative.

Notably, we also observed an upregulation of two genes involved in phosphatidylcholine (PC) synthesis (*pmt-1*, *pmt-2*)^84^ (Fig. 5). Intriguingly, PC was more abundant in the anoxic symbiont^16^, although the latter cannot synthetize it. Thus, their upregulation in AS worms suggests worm-to-symbiont lipid transfer.

#### GABA- and glutamate-mediated neurotransmission

**Figure 6:**
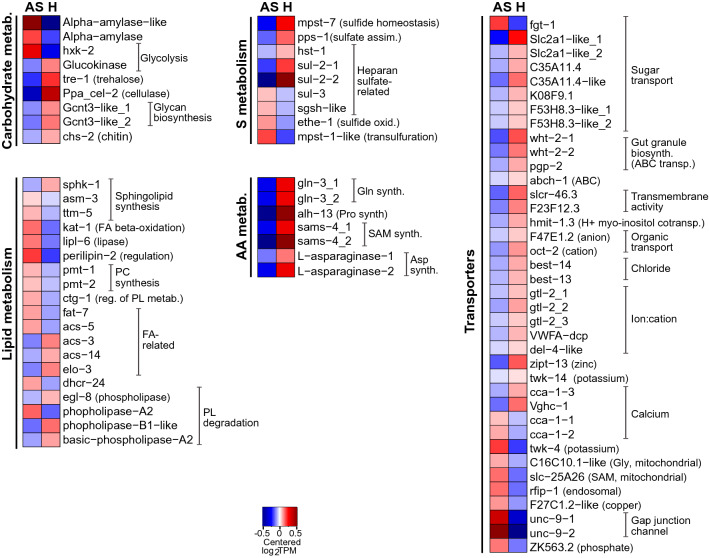
Genes involved in carbohydrate, lipid- and sulfur-metabolism, amino acids biosynthesis, and transport were predominantly expressed in hypoxic (H) worms. Heatmap displaying genes upregulated in H relative to AS worms, upon 24 h-long incubations under one of the two conditions (1.5-fold change, FDR ≤ 0.05). Expression levels are displayed as mean-centered log_2_TPM values (transcripts per kilobase million). Genes are ordered by function in their respective metabolic pathways. For each process, the minority of genes that were upregulated in AS worms is shown in Data S1. Red denotes upregulation, and blue downregulation. FA, fatty acids; PC phosphatidylcholine; PL, phospholipids; metab: metabolism; synth: synthesis; assim: assimilation, oxid: oxidation; Transp: transporters.

Upregulated genes related to GABA synthesis were *unc-25*, *unc-104* and *pdxk-1* (pyridoxal phosphate hexokinase)^85–87^ (Fig. 5, Data S1). Consistent with an expected increase in glutamate requirement as a direct GABA precursor^88^, we observed downregulation of two glutamine synthetases and a delta-1-pyrroline-5-carboxylate synthase (*gln-3* and *alh-13* respectively*;* Fig. 6)^89,90^, known to convert glutamate to glutamine or to proline, respectively. Furthermore, an *mgl-2*-like gene encoding for a glutamate receptor, which is activated in the presence of glutamate^91^, was up in AS worms. Note that, when oxygen is limited, glutamate may act as a neurotoxic amino acid^92^. Therefore, increased GABA biosynthesis might, beneficially, prevent its accumulation^93^.

GABA-mediated neurotransmission has been documented for facultative anaerobic animals thriving in anoxic conditions^92,94^. Due to its inhibitory nature, it contributes to avoid membrane depolymerization^94^. Moreover, given that it relaxes muscles, the increment of GABA may impact the movement of the animal^85^. Therefore, upregulation of GABA-mediated neuronal activity might explain why *L. oneistus* did not form tight clusters  in anoxia (Supplemental Movie 3).

#### Dopamine-mediated neurotransmission

A gene encoding for the tyrosine hydroxylase Cat-2 (*cat-2*), which is needed for dopamine biosynthesis^95^ and two putative dopamine receptors (protein-D2-like and a G_PROTEIN_RECEP_F1_2 domain-containing protein (*dop-5*))^96^ were upregulated in AS worms. Moreover, a *dat-1*-like gene mediating dopamine reuptake into the presynaptic terminals was downregulated^97^ in AS worms (Fig. 5).

#### Calcium-binding and -sensing proteins

Finally, in AS worms several calcium-binding or -sensing proteins (e.g., *ncs-2*, *cex-2*, and a calbindin-like (CALB1 homolog); Fig. 5)^98,99^, as well as calcium transporters (*cca-1*; Transport category, Fig. 6)^100^ were upregulated. On the one hand, we hypothesize their involvement in the inhibitory neural signaling described above (for example, Ncs-2 mediates the cholinergic and GABAergic expression of *C. elegans*^101^. On the other, they may protect cells against the stress inflicted by anoxia, which involves calcium overload and consequent cellular acidification^7^.

### Genes upregulated in hypoxic (H) nematodes

#### Innate immune pathways and effectors

Animals recognize and respond to microbes by means of immunoreceptors including Toll-like receptors, conserved from sponges to humans^102^. We identified almost all genes belonging to this pathway, including the one encoding for the NF-kB transcription factor. This came as a surprise given that, up to now, the NF-kB has not been identified in any other nematode^103^. Equally surprising was the fact that not only two Toll-like receptors (*tol-1* and *tol-1-like*), but also genes encoding for antimicrobial proteins such as a peroxisome assembly factor involved in defense against Gram- (*prx-11*-like)^104^, a putatively antifungal endochitinase^105^ and bactericidal/permeability-increasing proteins (BPIs) were also more abundant in H worms. BPIs may bind LPS and perforate Gram- membranes and have been shown to play a symbiostatic role in other invertebrates^106,107^. However, it is unclear whether activation of the *L. oneistus* Toll pathway leads to the nuclear NF-kB switching on the expression of antimicrobial genes or whether, as shown in *C. elegans*, the Toll pathway mediates behavioral avoidance of pathogens^108^.

Overall, the apparent oxygen stimulation of a central innate immunity pathway and, directly or indirectly, of broad range antimicrobials could be adaptations to the fact that when crawling in superficial oxygenated sand, *L. oneistus* is not only exposed to predation by bigger animals, but also to pathogenic members of the bacterioplankton. Overexpression of broad-range antimicrobials in response to oxygen might therefore help *L. oneistus* to avoid colonization by potentially deleterious, fouling bacteria (e.g., *vbrios*, *roseobacters* and *pseudoaltermonas/alteromonadales*) when crawling close to the water column^109^ (M. Mussmann, personal communication).

#### Development

Although development-related genes were some of the most expressed under all conditions (Fig. 1), many were upregulated in H nematodes (Figs. 2 and 5). Among the development-related genes upregulated in H nematodes were those related to molting (e.g., *nas-36*, *nas-38*, *chs-2*, *ptr-5*, *ptr-18*, *apl-1*, *myrf-1*)^110–114^, germ line establishment (e.g., *ccm-3*, *rsks-1*)^115,116^, oogenesis/spermatogenesis (*crt-1*)^117^, embryonic development and yolk production (*e.g., plt-1*, *mlc-5, crt-1*, *arrd-17*, *cpna-1*, *vit-6*)^118–123^, and/or larval development (*nmy-1*, *ifb-1*)^124,125^, as well as male tail tip (*cdt-1*, *plx-1*, *ver-3*)^126–128^, and vulva morphogenesis (*hda-1*, *unc-62*)^129,130^ (Fig. 5). Morever, transcripts encoding for a number of proteases shown to be involved in *C. elegans* molting (e.g., *nas-36*, *nas-38*)^110^, development (e.g., teneurin-a-like)^131^, neuronal regrowth or locomotion (*tep-1*)^132^ and pharyngeal pumping (e.g., neprilysin *nep-1*)^133^ were also more abundant in H worms. Remarkably, *vav-1*, which besides being involved in male tail tip and vulva morphogenesis^126^ may also regulate the concentration of intracellular calcium^134^, was one of the few development-related genes to be downregulated in H nematodes (see previous section on Ca^2+^-binding proteins).

To sum up, and as expected, the host appears to exploit oxygen availability to undertake energetically costly processes, such as development and molting^135^.

#### Carbohydrate metabolism

If in AS nematodes, glycogen or starch appeared prominent carbon sources, H worms seemed to exploit trehalose and cellulose instead. Indeed, genes that degrade trehalose (*tre-1*)^136^, and cellulose (Ppa-*cel-2*)^137^ were upregulated in H worms, as well as a putative ADP-dependent glucokinase (C50D2.7) involved in glycolysis^138^. The use of this pathway was supported by the overexpression of four genes encoding for sugar transporters (Slc2-A1, C35A11, K08F9.1, F53H8.3)^139,140^, perhaps switched on by active mTOR (see above) (Fig. 6)^69^.

Additionally, *L. oneistus* appeared to exploit oxygen to synthesize complex polysaccharides, such as heparan sulfate (*hst-1-*like)^141^ and glycan (*Gcnt*3-like) (Fig. 6), as an ortholog of the N-deacetylase/N-sulfotransferase *hst-1*, related to heparin biosynthesis was also upregulated^141^.

Although glycolysis seems to generate ATP in both AS and H worms, it is not clear why the latter would prefer to respire cellulose or trehalose instead of starch. Given its role as a membrane stabilizer, we speculate that AS worms might prioritize the storage of trehalose over its degradation to preserve membrane integrity (Fig. 6)^4,142^. Of note, based on its genome draft, the symbiont may synthetize and transport trehalose, but it may not use it^16^. Therefore, we hypothesize symbiont-to-host transfer of trehalose under hypoxia. Consistently, the symbiont’s trehalose synthesis-related gene (*otsB*)^16^, and the host trehalase (*tre-1*; Fig. 6) were both upregulated under hypoxia and metabolomics could detect trehalose in both partners (Table S1). Metabolomics also detected sucrose in both the holobiont and the symbiont fraction (Table S1). Given that, based on transcriptomics and proteomics, the nematode can utilize sucrose but cannot synthesize it (Data S1), whereas the symbiont can^16^, we also hypothesize symbiont-to-host sucrose transfer.

#### Acetylcholine-mediated neurotransmission

Instead of upregulating genes involved in inhibitory (GABA- and dopamine-mediated) neurotransmission, hypoxic worms appeared to use excitatory acetylcholine-mediated neurotransmission as indicated by the upregulation of *molo-1*, *acr-20,* cup-4, *lev-9*, and sphingosine kinase *sphk-1* that promotes its release^143–147^ (Fig. 5). On the one hand, acetylcholine-mediated neurotransmission might promote ROS detoxification in H worms^148^. On the other hand, its downregulation in AS worms may beneficially decrease calcium influx^3^.

#### Feeding, mating, mechanosensory behavior and axon guidance and fasciculation

Transcripts related to the neuronal regulation of energy-demanding activities such as feeding, mating, motion, as well as nervous system development were more abundant in H nematodes (Fig. 5, and Data S1). More precisely, upregulated genes were involved in pharyngeal pumping (*nep-1*, *lat-2*)^133,149^, male mating behavior and touch (*pdfr-1*, *tbb-4, ebax-1*)^150,151^, axon guidance and fasciculation (*spon-1*, *igcm-1*, *ebax-1*, *tep-1*)^132,151–153^, mechanosensory behavior (e.g., *mec-12*, *delm-2*)^154,155^. If we assume that *L. oneistus* mates in H conditions, genes involved in mechanosensory behavior may be upregulated for the male to find the female organ (vulva). Additionally, we also observed the upregulation of a gene encoding for a glutamate receptor (*glr-7*) possibly involved in feeding facilitation^156^.

#### Amino acid biosynthesis

Transcripts of genes involved in the synthesis of glutamine and proline (*gln-3* and *alh-13*, respectively), aspartate (L-asparaginases)^157^ and S-adenosyl-L-methionine (SAM) (*sams-4*)^158^ were all upregulated in H worms (Fig. 6), as well as one encoding for the ornithine decarboxylase *odc-1* which is involved in biosynthesis of the polyamine putrescin, and is essential for cell proliferation and tissue growth^159^. Moreover, polyamines, with their high charge-to-mass ratio may protect against superoxide radicals, which, as mentioned, harm cell membranes and organelles, oxidize proteins, and damage DNA^160^.

#### Lipid biosynthesis

Genes upregulated in H worms mediate the biosynthesis of long chain fatty acids (*acs-3*, *acs-14*, *elo-*3 but not *acs-5*)^138,161,162^, sphingolipids (a sphingosine kinase-1 (*sphk-1*) and *egl-8*, which controls egg laying and pharyngeal pumping in *C. elegans*^163^. Notably, sphingolipids may be anti-apoptotic^164^ or result in acetylcholine release^147^.

On the other hand, ceramides, which have anti-proliferative properties and which may mediate resistance to severe oxygen deprivation^165^, appeared to be mainly synthesized in AS worms, as indicated by the upregulation of genes involved in ceramide biosynthesis (*asm-3*, *ttm-5*, Fig. 6)^166^.

#### Transport

As anticipated in the introduction, anoxia-tolerant animals switch off ATP-demanding processes such as ion pumping^7^. Indeed, transcripts encoding for proteins involved in cation channel activity (*gtl-2*, voltage gated H channel 1)^167^, sodium transport (*delm-2*-like)^155^, chloride transport (*anoh-1*, *best-13*, *best-14*)^168–170^, ABC transporters (*wht-2*, *pgp-2*, slcr-46.3, F23F12.3, *hmit-1.3*)^171–173^ and organic substance transport (F47E1.2, *oct-2*)^174^ were more abundant in H than AS worms (Fig. 6).

#### Sulfur metabolism

The *mpst-7* gene, involved in organismal response to selenium and switched on in hypoxic *C. elegans*^175^, was upregulated in H nematodes (Fig. 6). Given that MPST-7 is thought to catalyze the conversion of sulfite and glutathione persulfide (GSSH) to thiosulfate and glutathione (GSH)^176^, hypoxia-experiencing *L. oneistus* might express this enzyme to recharge the cells with GSH and hence, help to cope with oxidative stress^177^. Also more abundant in H worms were transcripts encoding for the sulfatases 2 (*sul-*2)^178^ and a PAPS-producing *pps-1* (3′-phospho-adenosine-5′-phosphosulfate (PAPS), considered the universal sulfur donor)^141^, as well as for the chaperones *pdi-6* and protein-disulfide-isomerase-A5-like which require oxygen to mediate correct disulfide bond formation in protein folding^6,179^ (Fig. 6). Conversely, a putative sulfide-producing enzyme (*mpst-1*) which protects *C. elegans* from mitochondrial damage^180^ was upregulated in AS nematodes, albeit together with two genes encoding for enzymes involved in its detoxification, a persulfide dioxygenase (*ethe-1*) and a cysteine dioxygenase (*cdo-1*)^179^. The first detoxifies sulfide by producing glutathione, while the latter catalyzes taurine synthesis via cysteine degradation. Of note, sulfide detoxification via taurine accumulation is a common strategy in chemosynthetic animals (reviewed in^181^).

All in all, *L. oneistus* appeared to limit excess accumulation of free sulfide in anoxia and to free sulfate when oxygen was available.

## Conclusions

**Figure 7:**
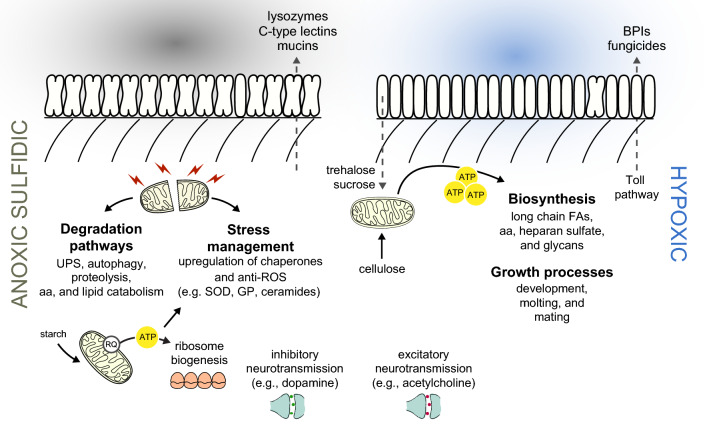
Schematic representation of *Laxus oneistus* physiology in anoxic or hypoxic  conditions. In anoxic sulfidic  conditions (left), *L. oneistus* does not enter suspended animation. Instead, it upregulates the expression of genes mediating inhibitory neurotransmission, involved in symbiosis establishment (e.g., lectins, mucins) and in ribosome biogenesis. Metabolism may be supported by the degradation of starch and by rewiring the electron transfer chain: rhodoquinone (RQ) is used as electron carrier and fumarate as electron acceptor. Moreover, the worm activates degradation pathways (e.g., ubiquitin–proteasome system (UPS), autophagy, and apoptosis) and may anticipate reoxygenation by upregulating superoxide dismutase (SOD) and glutathione peroxidase (GP). In hypoxic  conditions (right), instead, *L. oneistus* appears to use trehalose and cellulose for energy generation, while engaging in costly processes such as development, molting, feeding, and mating. Genes involved in excitatory neurotransmission are also upregulated, together with Toll-like receptors and immune effectors (e.g., fungicides, BPIs).

Overall, and irrespective of the conditions it was subjected to, *L. oneistus* mostly expressed genes involved in degradation processes, energy generation, stress response and immune defense. Astonishingly, *L. oneistus* did not enter suspended animation when subjected to anoxic sulfidic conditions for days. We hypothesize that in the absence of oxygen, ATP production is supported by symbiont-derived trehalose and cellulose catabolism, and by rewiring the ETC in such way as to use RQ as electron carrier, and fumarate as electron acceptor. Moreover, the nematode activates several degradation pathways (e.g., UPS, autophagy, and apoptosis) to gain nutrients from anoxia-damaged proteins and mitochondria. Further, AS worms also upregulated genes encoding for ribosomal proteins and putative symbiont-binding proteins (lectins). Finally, as proposed for other anoxia-tolerant animals, the worm seems to upregulate its antioxidant capacity in anticipation of reoxygenation. When in hypoxic conditions (Fig. 7, left), instead, we speculate that the worm uses starch for energy generation to engage in costly developmental processes such as molting, feeding, and mating, likely relying on excitatory neurotransmitters (e.g., acetylcholine), and it upregulates the Toll immune pathway and, directly or indirectly, the synthesis of broad range antimicrobials (e.g., fungicides, BPIs).

When looking at the *Laxus*-*Thiosymbion* symbiosis in light of what was recently published^16^, we could identify two signs of inter-partner metabolic dependence: under anoxia, worms might transfer lipids to their symbionts, and under hypoxia, the symbionts might transfer trehalose and sucrose to their hosts.

Furthermore, we may conclude that, wherever in the sand the consortium is, one of the two partners is bound to be stressed: in anoxia, the symbiont appears to proliferate more, while its animal host engages in degradation of damaged proteins and mitochondria and in detoxification. In the presence of oxygen, the situation is inverted: the symbiont seems massively stressed, while the host can afford energy-costly biosynthetic processes to develop and reproduce (Fig. 7). It is therefore fascinating that, in spite of the dramatically different needs a bacterium and animal must have, the *Laxus*-*Thiosymbion* symbiosis evolved.

## Materials and methods

### Sample collection

*Laxus oneistus* individuals were collected on multiple field trips (2016–2019) with cores of 60 cm length and 60 mm diameter (UWITEC, Mondsee, Austria) down to a depth of approximately 1 m depth in the sand bars off the Smithsonian Field Station, Carrie Bow Cay in Belize (16°48′11.01″N, 88°4′54.42″W). The collection of the nematodes, the incubations set up for RNA sequencing, lipidomics, proteomics and metabolomics, as well as the RNA extraction, and library preparation are in detailed described in^16^. Importantly, the nematodes had a bright white appearance and replicate incubations were started simultaneously. Note that the Supplemental material describes the metabolomics and sequencing data of *Olavius algarvensis*, as well as changes from^16^ in the lipidomics and proteomics pipelines.

### Host transcriptome de novo assembly

In preparation for the assembly, reads from each sample were first mapped to the symbiont as described before^16^, and remaining rRNA reads from all domains of life were removed from unmapped reads using sortmerna v2.1 in combination with the SSURef_NR99_119_SILVA_14_07_14 and LSURef_119_SILVA_15_07_14 databases. Further, exact duplicate reads were removed using PRINSEQ lite’s derep option. Read files free of symbiont reads, rRNA reads and exact duplicates were used as input for transcriptome sub-assemblies via Trinity v2.6.6 with the strand-specific option (--SS_lib_type F)^182^. Two sub-assemblies differing in the number and type of input read files were performed: (1) 9 input read files including biological triplicates from 3 incubation conditions (O, H, A) and (2) 4 input read files including a single replicate from 4 incubation conditions (O, H, A and hyper-O). Hyper-O refers to an incubation in which air was pumped directly into the exetainers for the entire incubation period to supersaturate the seawater (300% O_2_). However, as this incubation condition yielded an incongruous transcriptional response by the symbiont (data not shown), these read data were only used to extend the host transcriptome’s coding repertoire. The qualities of both sub-assemblies were assessed as described below.

We then performed an intra-assembly clustering step as described in^183^, during which identical transcripts were removed from the sub-assemblies using CD-HIT-EST^184^. To further reduce redundant transcripts, only the longest isoform for each ‘gene’ identified by Trinity was kept using Trinity’s get_longest_isoform_seq_per_trinity_gene.pl utility. The remaining transcripts of each sub-assembly were then concatenated to produce a merged transcriptome assembly. The final assembly was created by applying another sequence clustering using CD-HIT-EST to avoid inter-assembly redundancy. Here, the identity parameter of 80% (-c 0.8) combined with a minimal coverage ratio of the shorter sequence of 80% (-aS 0.8) and minimal coverage ratio of the longest sequence of 0.005% (-aL 0.005) yielded the best-performing assembly in terms of number of transcripts (162,455) and contiguity (N50 value of 770) (data not shown).

Assembly completeness was assessed by estimating completeness via BUSCO nematode single-copy orthologs (Simão et al. 2015). Importantly, the merged assembly yielded a higher BUSCO-based completeness compared with the two sub-assemblies; 79.2% of the BUSCO nematode single-copy orthologs were found to be present and complete in the final assembly (636 single-copy; 142 duplicated), whereas assembly (1) scored 77.8% (233 single-copy; 531 duplicated) and assembly (2) was 76.2% complete (314 single-copy; 434 duplicated). Further, assembled transcripts were filtered based on taxonomic classification. Transcripts were matched against the RefSeq protein database using blastx (E-value 1E−3), and the output was then used as input for taxonomic assignment via MEGAN v5^185^. Only transcripts classified as belonging to ‘Eukarya’ were kept (MEGAN parameters: Min Score: 50, Max Expected: 1E-2, Top Percent: 2), which reduced the number of putative *L. oneistus* transcripts to 30,562. Assembled transcripts were also functionally annotated using Trinotate^186^. Briefly, predicted protein coding regions were extracted using TransDecoder (https://github.com/TransDecoder), both transcripts and predicted protein sequences were searched for protein homology via blastx and blastp, respectively, and predicted protein sequences were annotated for protein domains (hmmscan), signal peptides (signalP) and transmembrane domains (TMHMM). 85,859 transcripts exhibited at least one functional annotation. Finally, only taxonomy-filtered transcripts with at least one functional annotation were kept, thereby further reducing the number of putative host transcripts to 27,984, with 22,072 thereof predicted to contain protein coding regions. BUSCO-based completeness for this filtered host transcriptome assembly was 78.8% (635 single-copy; 139 duplicated).

### Gene expression analysis

Raw sequencing reads quality assessment and preprocessing of data was followed as described in^16^. Trimmed reads were mapped to the de novo transcriptome assembly and transcript abundance was estimated using RSEM v1.3.1^187^ in combination with bowtie2 with default settings except for the application of strandedness (--strandedness forward). Read counts per transcript were used for differential expression analysis, and TPM (transcripts per kilobase million) values were transformed to log_2_TPMs as described in^16^.

Gene and differential expression analyses were conducted using R and the Bioconductor package edgeR v3.28.1^188,189^, and as shown in^16^. Here, we only describe the modifications that were made to the pipeline. Genes were considered expressed if at least ten reads in at least three replicates of one of the four conditions could be assigned. Excluding the replicates of the oxic condition, we found 74.9% of all predicted nematode protein-encoding genes to be expressed (16,526 genes out of 22,072). Log_2_TPM were used to assess sample similarities via multidimensional scaling based on Euclidean distances (R Stats package)^189^ (Fig. S1B), and the average of replicate log_2_TPM values per expressed gene and condition was used to estimate expression strength. Median gene expression of entire metabolic processes and pathways per condition was determined from average log_2_TPM values.

Expression of genes was considered significantly different if their expression changed 1.5-fold between two treatments with a false-discovery rate (FDR) ≤ 0.05^190^. Throughout the paper, all genes meeting these thresholds are either termed differentially expressed or up- or downregulated. For the differential expression analyses between the AS, H and A conditions see Data S1. Heatmaps show mean-centered log_2_TPM expression values to highlight gene expression change.

All predicted *L. oneistus* proteins were automatically annotated using eggNOG-mapper v2^191^ against eggNOG 5.0^192^ using diamond v2.0.4^193^. All genes that are shown and involved in a particular process were manually curated by blasting (blastp) them against both the nr database^194^ and the WormBase (https://wormbase.org/tools/blast_blat)^195^. The Spearman’s rank correlation was carried out with per-gene averaged log_2_TPM and orgNSAF% values over the oxic and anoxic incubations for the transcriptome and proteome, respectively, in R^189^.

## Supplementary Information

Supplementary Information 1.

Supplementary Information 2.

Supplementary Figure S1.

Supplementary Figure S2.

Supplementary Figure S3.

Supplementary Figure S4.

Supplementary Figure S5.

Supplementary Information 3.

Supplementary Movie 1.

Supplementary Movie 2.

Supplementary Movie 3.

Supplementary Movie 4.

Supplementary Legends.

Supplementary Table S1.

## Data Availability

This Transcriptome Shotgun Assembly project has been deposited at DDBJ/EMBL/GenBank under the accession GJNO00000000. The version described in this paper is the first version, GJNO01000000. RNA-Seq data are available at the Gene Expression Omnibus (GEO) database and are accessible through accession number GSE188619.

## References

[1] Hermes-Lima M, Zenteno-Savín T (2002). Animal response to drastic changes in oxygen availability and physiological oxidative stress. Comp. Biochem. Physiol. C.

[2] Hochachka PW, Buck LT, Doll CJ, Land SC (1996). Unifying theory of hypoxia tolerance: Molecular/metabolic defense and rescue mechanisms for surviving oxygen lack. Proc. Natl. Acad. Sci. U. S. A..

[3] Hochachka PW, Lutz PL (2001). Mechanism, origin, and evolution of anoxia tolerance in animals. Comp. Biochem. Physiol. B.

[4] Clegg JS (1997). Embryos of Artemia franciscana survive four years of continuous anoxia: The case for complete metabolic rate depression. J. Exp. Biol..

[5] Nystul TG, Goldmark JP, Padilla PA, Roth MB (2003). Suspended Animation in *C. elegans* Requires the Spindle Checkpoint. Science.

[6] Teodoro RO, O’Farrell PH (2003). Nitric oxide-induced suspended animation promotes survival during hypoxia. EMBO J..

[7] Galli GLJ, Richards JG (2014). Mitochondria from anoxia-tolerant animals reveal common strategies to survive without oxygen. J. Comp. Physiol. B.

[8] Padilla PA, Nystul TG, Zager RA, Johnson ACM, Roth MB (2002). Dephosphorylation of cell cycle-regulated proteins correlates with anoxia-induced suspended animation in *Caenorhabditis elegans*. Mol. Biol. Cell.

[9] Powell-Coffman JA (2010). Hypoxia signaling and resistance in *C. elegans*. Trends Endocrinol. Metab..

[10] Kitazume H, Dayi M, Tanaka R, Kikuchi T (2018). Assessment of the behaviour and survival of nematodes under low oxygen concentrations. PLoS ONE.

[11] Nystul TG, Roth MB (2004). Carbon monoxide-induced suspended animation protects against hypoxic damage in *Caenorhabditis elegans*. Proc. Natl. Acad. Sci. U. S. A..

[12] Kim KW, Jin Y (2015). Neuronal responses to stress and injury in *C. elegans*. FEBS Lett..

[13] Denny MW (1993). Air and Water: The Biology and Physics of Life’s Media.

[14] Ott JA (1991). Tackling the sulfide gradient: A novel strategy involving marine nematodes and chemoautotrophic ectosymbionts. Mar. Ecol..

[15] Schiemer F, Novak R, Ott J (1990). Metabolic studies on thiobiotic free-living nematodes and their symbiotic microorganisms. Mar. Biol..

[16] Paredes GF (2021). Anaerobic sulfur oxidation underlies adaptation of a chemosynthetic symbiont to oxic-anoxic interfaces. mSystems.

[17] Ott JA, Novak R (1989). Living at an interface: Meiofauna at the oxygen/sulfide boundary of marine sediments. Reprod. Genet. Distrib. Mar..

[18] Ott JA, Bauer-Nebelsick M, Novotny V (1995). The genus Laxus Cobb, 1894 (Stilbonematinae:Nematoda ): Description of two new species with ectosymbiotic chemoautotrophic bacteria. Proc. Biol. Soc. Wash..

[19] Bateman A (2021). UniProt: The universal protein knowledgebase in 2021. Nucleic Acids Res..

[20] Stringham EG, Jones D, Candido EPM (1992). Expression of the polyubiquitin-encoding gene (ubq-1) in transgenic *Caenorhabditis elegans*. Gene.

[21] Geuens E (2010). Globin-like proteins in *Caenorhabditis elegans*: In vivo localization, ligand binding and structural properties. BMC Biochem..

[22] Birnby DA (2000). A Transmembrane guanylyl cyclase (DAF-11) and Hsp90 (DAF-21) regulate a common set of chemosensory behaviors in *Caenorhabditis elegans*. Genetics.

[23] Chávez V, Mohri-Shiomi A, Maadani A, Vega LA, Garsin DA (2007). Oxidative stress enzymes are required for DAF-16-mediated immunity due to generation of reactive oxygen species by *Caenorhabditis elegans*. Genetics.

[24] Sun J (2017). Adaptation to deep-sea chemosynthetic environments as revealed by mussel genomes. Nat. Ecol. Evol..

[25] Hinzke T (2019). Host-microbe interactions in the chemosynthetic riftia pachyptila symbiosis. MBio.

[26] Yuen B, Polzin J, Petersen JM (2019). Organ transcriptomes of the lucinid clam *Loripes orbiculatus* (Poli, 1791) provide insights into their specialised roles in the biology of a chemosymbiotic bivalve. BMC Genom..

[27] Woyke T (2006). Symbiosis insights through metagenomic analysis of a microbial consortium. Nature.

[28] Wippler J (2016). Transcriptomic and proteomic insights into innate immunity and adaptations to a symbiotic lifestyle in the gutless marine worm *Olavius algarvensis*. BMC Genom..

[29] Zimmermann J (2016). Closely coupled evolutionary history of ecto- and endosymbionts from two distantly related animal phyla. Mol. Ecol..

[30] Naylor DJ, Hoogenraad NJ, Høj PB (1996). Isolation and characterisation of a cDNA encoding rat mitochondrial GrpE, a stress-inducible nucleotide-exchange factor of ubiquitous appearance in mammalian organs. FEBS Lett..

[31] Lundin VF, Srayko M, Hyman AA, Leroux MR (2008). Efficient chaperone-mediated tubulin biogenesis is essential for cell division and cell migration in *C. elegans*. Dev. Biol..

[32] Bar-Lavan Y (2016). A differentiation transcription factor establishes muscle-specific proteostasis in *Caenorhabditis elegans*. PLOS Genet..

[33] Margis R, Dunand C, Teixeira FK, Margis-Pinheiro M (2008). Glutathione peroxidase family: An evolutionary overview. FEBS J..

[34] Oliveira RP (2009). Condition-adapted stress and longevity gene regulation by *Caenorhabditis elegans* SKN-1/Nrf. Aging Cell.

[35] Murphy E, Steenbergen C (2008). Mechanisms underlying acute protection from cardiac ischemia-reperfusion injury. Physiol. Rev..

[36] Heo JM (2010). A stress-responsive system for mitochondrial protein degradation. Mol. Cell.

[37] Tullet JMA (2015). DAF-16 target identification in *C. elegans*: Past, present and future. Biogerontology.

[38] Kaushal PS (2014). Cryo-EM structure of the small subunit of the mammalian mitochondrial ribosome. Proc. Natl. Acad. Sci. U. S. A..

[39] Sharika R, Subbaiah P, Balamurugan K (2018). Studies on reproductive stress caused by candidate Gram positive and Gram negative bacteria using model organism, *Caenorhabditis elegans*. Gene.

[40] Melnikov S (2012). One core, two shells: Bacterial and eukaryotic ribosomes. Nat. Struct. Mol. Biol..

[41] You KT, Park J, Kim VN (2015). Role of the small subunit processome in the maintenance of pluripotent stem cells. Genes Dev..

[42] Savada RP, Bonham-Smith PC (2014). Differential transcript accumulation and subcellular localization of Arabidopsis ribosomal proteins. Plant Sci..

[43] Xu X, Xiong X, Sun Y (2016). The role of ribosomal proteins in the regulation of cell proliferation, tumorigenesis, and genomic integrity. Sci. China Life Sci..

[44] Larade K, Nimigan A, Storey KB (2001). Transcription pattern of ribosomal protein L26 during anoxia exposure in *Littorina littorea*. J. Exp. Zool..

[45] Xu C (2018). Genetic inhibition of an ATP synthase subunit extends lifespan in *C. elegans*. Sci. Rep..

[46] Maglioni S, Ventura N (2016). *C. elegans* as a model organism for human mitochondrial associated disorders. Mitochondrion.

[47] McKay RM, McKay JP, Avery L, Graff JM (2003). *C. elegans*: A Model for exploring the genetics of fat storage. Dev. Cell.

[48] Rea SL, Ventura N, Johnson TE (2007). Relationship between mitochondrial electron transport chain dysfunction, development, and life extension in *Caenorhabditis elegans*. PLOS Biol..

[49] Hartman PS, Ishii N, Kayser EB, Morgan PG, Sedensky MM (2001). Mitochondrial mutations differentially affect aging, mutability and anesthetic sensitivity in *Caenorhabditis elegans*. Mech. Ageing Dev..

[50] Williams JC (2005). Crystal structure of human SCO1: Implications for redox signaling by a mitochondrial cytochrome c oxidase ‘assembly’ protein. J. Biol. Chem..

[51] Roberts Buceta PM (2019). The kynurenine pathway is essential for rhodoquinone biosynthesis in *Caenorhabditis elegans*. J. Biol. Chem..

[52] Del Borrello S (2019). Rhodoquinone biosynthesis in *C. elegans* requires precursors generated by the kynurenine pathway. Elife.

[53] Tsai PC (2017). A recurrent WARS mutation is a novel cause of autosomal dominant distal hereditary motor neuropathy. Brain.

[54] Martínez-Reyes I, Chandel NS (2020). Mitochondrial TCA cycle metabolites control physiology and disease. Nat. Commun..

[55] Yang HC (2019). IDH-1 deficiency induces growth defects and metabolic alterations in GSPD-1-deficient *Caenorhabditis elegans*. J. Mol. Med..

[56] Jackson AD, McLaughlin J (2009). Digestion and absorption. Surgery.

[57] Papaevgeniou N, Chondrogianni N (2014). The ubiquitin proteasome system in *Caenorhabditis elegans* and its regulation. Redox Biol..

[58] Jones D, Crowe E, Stevens TA, Peter E, Candido M (2001). Functional and phylogenetic analysis of the ubiquitylation system in *Caenorhabditis elegans*: Ubiquitin-conjugating enzymes, ubiquitin-activating enzymes, and ubiquitin-like proteins. Genome Biol..

[59] Schaefer H, Rongo C (2006). KEL-8 is a substrate receptor for CUL3-dependent ubiquitin ligase that regulates synaptic glutamate receptor turnover. Mol. Biol. Cell.

[60] Brockway H, Balukoff N, Dean M, Alleva B, Smolikove S (2014). The CSN/COP9 signalosome regulates synaptonemal complex assembly during meiotic prophase I of *Caenorhabditis elegans*. PLOS Genet..

[61] Syntichaki P, Xu K, Driscoll M, Tavernarakis N (2002). Specific aspartyl and calpain proteases are required for neurodegeneration in *C. elegan*s. Nature.

[62] Fischer M, Fitzenberger E, Kull R, Boll M, Wenzel U (2014). The zinc matrix metalloproteinase ZMP-2 increases survival of *Caenorhabditis elegans* through interference with lipoprotein absorption. Genes Nutr..

[63] Wang RC, Levine B (2010). Autophagy in cellular growth control. FEBS Lett..

[64] Russell RC, Yuan HX, Guan KL (2014). Autophagy regulation by nutrient signaling. Cell Res..

[65] Liang XH (1999). Induction of autophagy and inhibition of tumorigenesis by beclin 1. Nature.

[66] Bar-Peled L, Schweitzer LD, Zoncu R, Sabatini DM (2012). Ragulator is a GEF for the rag GTPases that signal amino acid levels to mTORC1. Cell.

[67] Thompson AR, Vierstra RD (2005). Autophagic recycling: Lessons from yeast help define the process in plants. Curr. Opin. Plant Biol..

[68] Iadevaia V, Liu R, Proud CG (2014). MTORC1 signaling controls multiple steps in ribosome biogenesis. Semin. Cell Dev. Biol..

[69] Howell JJ, Manning BD (2011). MTOR couples cellular nutrient sensing to organismal metabolic homeostasis. Trends Endocrinol. Metab..

[70] Huber LA, Teis D (2016). Lysosomal signaling in control of degradation pathways. Curr. Opin. Cell Biol..

[71] Nussbaumer AD, Bright M, Baranyi C, Beisser CJ, Ott JA (2004). Attachment mechanism in a highly specific association between ectosymbiotic bacteria and marine nematodes. Aquat. Microb. Ecol..

[72] Bulgheresi S (2011). Sequence variability of the pattern recognition receptor Mermaid mediates specificity of marine nematode symbioses. ISME J..

[73] Bulgheresi S (2006). A new C-type lectin similar to the human immunoreceptor DC-SIGN mediates symbiont acquisition by a marine nematode. Appl. Environ. Microbiol..

[74] Koropatkin NM, Cameron EA, Martens EC (2012). How glycan metabolism shapes the human gut microbiota. Nat. Rev. Microbiol..

[75] Kaufmann SH (2008). Apoptosis-associated caspase activation assays. Methods.

[76] Chen YZ, Mapes J, Lee ES, Robert Skeen-Gaar R, Xue D (2013). Caspase-mediated activation of *Caenorhabditis elegans* CED-8 promotes apoptosis and phosphatidylserine externalization. Nat. Commun..

[77] Samejima K, Earnshaw WC (2005). Trashing the genome: The role of nucleases during apoptosis. Nat. Rev. Mol. Cell Biol..

[78] Sasaki A (2013). Arl8/ARL-8 functions in apoptotic cell removal by mediating phagolysosome formation in *Caenorhabditis elegans*. Mol. Biol. Cell.

[79] Hurwitz ME (2009). Abl kinase inhibits the engulfment of apopotic cells in *Caenorhabditis elegans*. PLoS Biol..

[80] Berdichevsky A (2010). 3-Ketoacyl thiolase delays aging of *Caenorhabditis elegans* and is required for lifespan extension mediated by sir-2.1. Proc. Natl. Acad. Sci. U. S. A..

[81] Chughtai AA (2015). Perilipin-related protein regulates lipid metabolism in *C. elegans*. PeerJ.

[82] Horikawa M, Sakamoto K (2009). Fatty-acid metabolism is involved in stress-resistance mechanisms of *Caenorhabditis elegans*. Biochem. Biophys. Res. Commun..

[83] Krivoruchko A, Storey KB (2015). Turtle anoxia tolerance: Biochemistry and gene regulation. Biochim. Biophys. Acta.

[84] Brendza KM (2007). Phosphoethanolamine N-methyltransferase (PMT-1) catalyses the first reaction of a new pathway for phosphocholine biosynthesis in *Caenorhabditis elegans*. Biochem. J..

[85] Mclntire SL, Jorgensen E, Kaplan J, Horvitz HR (1993). The GABAergic nervous system of *Caenorhabditis elegans*. Nature.

[86] Gally C, Bessereau JL (2003). GABA is dispensable for the formation of junctional GABA receptor clusters in *Caenorhabditis elegans*. J. Neurosci..

[87] Nordquist SK, Smith SR, Pierce JT (2018). Systematic functional characterization of human 21st chromosome orthologs in *Caenorhabditis elegans*. G3.

[88] Martin DL, Rimvall K (1993). Regulation of γ-aminobutyric acid synthesis in the brain. J. Neurochem..

[89] Van Der Vos KE, Coffer PJ (2012). Glutamine metabolism links growth factor signaling to the regulation of autophagy. Autophagy.

[90] Yen CA, Curran SP (2021). Incomplete proline catabolism drives premature sperm aging. Aging Cell.

[91] Tharmalingam S, Burns AR, Roy PJ, Hampson DR (2012). Orthosteric and allosteric drug binding sites in the *Caenorhabditis elegans* mgl-2 metabotropic glutamate receptor. Neuropharmacology.

[92] Lutz PL, Nilsson GE, Prentice HM (2002). The Brain Without Oxygen.

[93] Mathews GC, Diamond JS (2003). Neuronal glutamate uptake contributes to GABA synthesis and inhibitory synaptic strength. J. Neurosci..

[94] Milton SL, Lutz PL (1998). Low extracellular dopamine levels are maintained in the anoxic turtle (*Trachemys scripta*) striatum. J. Cereb. Blood Flow Metab..

[95] Sawin ER, Ranganathan R, Horvitz HR (2000). *C. elegans* locomotory rate is modulated by the environment through a dopaminergic pathway and by experience through a serotonergic pathway. Neuron.

[96] Sanyal S (2004). Dopamine modulates the plasticity of mechanosensory responses in *Caenorhabditis elegans*. EMBO J..

[97] McDonald PW, Jessen T, Field JR, Blakely RD (2006). Dopamine signaling architecture in *Caenorhabditis elegans*. Cell. Mol. Neurobiol..

[98] Soontornniyomkij V (2012). Hippocampal calbindin-1 immunoreactivity correlate of recognition memory performance in aged mice. Neurosci. Lett..

[99] Hobert O (2010). Neurogenesis in the nematode *Caenorhabditis elegans*. WormBook.

[100] Steger KA, Shtonda BB, Thacker C, Snutch TP, Avery LT (2005). *C. elegans* T-type calcium channel CCA-1 boosts neuromuscular transmission. J. Exp. Biol..

[101] Zhou K, Cherra SJ, Goncharov A, Jin Y (2017). Asynchronous cholinergic drive correlates with excitation-inhibition imbalance via a neuronal Ca2+ sensor protein. Cell Rep..

[102] Akira S, Uematsu S, Takeuchi O (2006). Pathogen recognition and innate immunity. Cell.

[103] Pujol N, Ewbank JJ (2021). *C. elegans*: Out on an evolutionary limb. Immunogenet..

[104] Wang D (2019). Epidermal barrier for nematodes against toxicity of environmental toxicants or stresses. Target Organ Toxicol. Caenorhabditis elegans.

[105] Dravid P, Kaushal DC, Saxena JK, Kaushal NA (2015). Isolation and characterization of endochitinase and exochitinase of *Setaria cervi*. Parasitol. Int..

[106] Krasity BC (2015). Structural and functional features of a developmentally regulated lipopolysaccharide-binding protein. MBio.

[107] Chen F (2017). Bactericidal permeability-increasing proteins shape host-microbe interactions. MBio.

[108] Brandt JP, Ringstad N (2015). Toll-like receptor signaling promotes development and function of sensory neurons required for a *C. elegans* pathogen-avoidance behavior. Curr. Biol..

[109] Dang H, Lovell CR (2016). Microbial surface colonization and biofilm development in marine environments. Microbiol. Mol. Biol. Rev..

[110] Suzuki M, Sagoh N, Iwasaki H, Inoue H, Takahashi K (2004). Metalloproteases with EGF, CUB, and thrombospondin-1 domains function in molting of *Caenorhabditis elegans*. Biol. Chem..

[111] Zhang Y, Foster JM, Nelson LS, Ma D, Carlow CKS (2005). The chitin synthase genes chs-1 and chs-2 are essential for *C. elegans* development and responsible for chitin deposition in the eggshell and pharynx, respectively. Dev. Biol..

[112] Zugasti O, Rajan J, Kuwabara PE (2005). The function and expansion of the Patched- and Hedgehog-related homologs in *C. elegans*. Genome Res..

[113] Hornsten A (2007). APL-1, a *Caenorhabditis elegan*s protein related to the human β-amyloid precursor protein, is essential for viability. Proc. Natl. Acad. Sci. U. S. A..

[114] Russel S, Frand AR, Ruvkun G (2011). Regulation of the *C. elegans* molt by pqn-47. Dev. Biol..

[115] Pan KZ (2007). Inhibition of mRNA translation extends lifespan in *Caenorhabditis elegans*. Aging Cell.

[116] Pal S (2017). CCM-3 promotes *C. elegans* germline development by regulating vesicle trafficking cytokinesis and polarity. Curr. Biol..

[117] Park BJ (2001). Calreticulin, a calcium-binding molecular chaperone, is required for stress response and fertility in *Caenorhabditis elegans*. Mol. Biol. Cell.

[118] Goedert M (1996). PTL-1, a microtubule-associated protein with tau-like repeats from the nematode *Caenorhabditis elegans*. J. Cell Sci..

[119] Gally C (2009). Myosin II regulation during *C. elegans* embryonic elongation:LET-502/ROCK, MRCK-1 and PAK-1, three kinases with different roles. Development.

[120] Zahreddine H, Zhang H, Diogon M, Nagamatsu Y, Labouesse M (2010). CRT-1/Calreticulin and the E3 Ligase EEL-1/HUWE1 control hemidesmosome maturation in *C. elegans* development. Curr. Biol..

[121] Jee C (2012). CNP-1 (ARRD-17), a novel substrate of calcineurin, Is critical for modulation of egg-laying and locomotion in response to food and lysine sensation in *Caenorhabditis elegans*. J. Mol. Biol..

[122] Warner A (2013). CPNA-1, a copine domain protein, is located at integrin adhesion sites and is required for myofilament stability in *Caenorhabditis elegans*. Mol. Biol. Cell.

[123] Perez MF, Lehner B (2019). Vitellogenins: Yolk gene function and regulation in *Caenorhabditis elegans*. Front. Physiol..

[124] Ding M, Woo WM, Chisholm AD (2004). The cytoskeleton and epidermal morphogenesis in *C. elegans*. Exp. Cell Res..

[125] Osório DS (2019). Crosslinking activity of non-muscle myosin II is not sufficient for embryonic cytokinesis in *C. elegans*. Development.

[126] Nelson MD (2011). A bow-tie genetic architecture for morphogenesis suggested by a genome-wide RNAi screen in *Caenorhabditis elegans*. PLOS Genet..

[127] Dalpé G, Zhang LW, Zheng H, Culotti JG (2004). Conversion of cell movement responses to Semaphorin-1 and Plexin-1 from attraction to repulsion by lowered levels of specific RAC GTPases in *C. elegans*. Development.

[128] Dalpe G, Tarsitano M, Graziella Persico M, Zheng H, Culotti J (2013). *C. elegans* PVF-1 inhibits permissive UNC-40 signalling through CED-10 GTPase to position the male ray 1 sensillum. Development.

[129] Dufourcq P (2002). Functional Requirement For Histone Deacetylase 1 in *Caenorhabditis elegans* gonadogenesis. Mol. Cell. Biol..

[130] Yang L, Sym M, Kenyon C (2005). The roles of two *C. elegans* HOX co-factor orthologs in cell migration and vulva development. Development.

[131] Topf U, Drabikowski K (2019). Ancient function of teneurins in tissue organization and neuronal guidance in the nematode *Caenorhabditis elegans*. Front. Neurosci..

[132] Kim KW (2018). Expanded genetic screening in *Caenorhabditis elegans* identifies new regulators and an inhibitory role for NAD + in axon regeneration. Elife.

[133] Spanier B, Stürzenbaum SR, Holden-Dye LM, Baumeister R (2005). *Caenorhabditis elegans* neprilysin NEP-1: An effector of locomotion and pharyngeal pumping. J. Mol. Biol..

[134] Norman KR (2005). The Rho/Rac-family guanine nucleotide exchange factor VAV-1 regulates rhythmic behaviors in *C. elegans*. Cell.

[135] Uppaluri S, Brangwynne CP (2015). A size threshold governs *Caenorhabditis elegans* developmental progression. Proc. R. Soc. B.

[136] Pellerone FI (2003). Trehalose metabolism genes in *Caenorhabditis elegans* and filarial nematodes. Int. J. Parasitol..

[137] Schuster LN, Sommer RJ (2012). Expressional and functional variation of horizontally acquired cellulases in the nematode *Pristionchus pacificus*. Gene.

[138] Yuan Y (2012). Enhanced energy metabolism contributes to the extended life span of calorie-restricted *Caenorhabditis elegans*. J. Biol. Chem..

[139] Kitaoka S, Morielli AD, Zhao FQ (2013). FGT-1 Is a mammalian GLUT2-like facilitative glucose transporter in *Caenorhabditis elegans* whose malfunction induces fat accumulation in intestinal cells. PLoS ONE.

[140] Bertoli S (2015). Short-term effects of ketogenic diet on anthropometric parameters, body fat distribution, and inflammatory cytokine production in GLUT1 deficiency syndrome. Nutrition.

[141] Bhattacharya R, Townley RA, Berry KL, Bülow HE (2009). The PAPS transporter PST-1 is required for heparan sulfation and is essential for viability and neural development in *C. elegans*. J. Cell Sci..

[142] Chen Q, Enbo MA, Behar KL, Xu T, Haddad GG (2002). Role of trehalose phosphate synthase in anoxia tolerance and development in *Drosophila melanogaster**. J. Biol. Chem..

[143] Mongan NP, Jones AK, Smith GR, Sansom MSP, Sattelle DB (2002). Novel α7-like nicotinic acetylcholine receptor subunits in the nematode *Caenorhabditis elegans*. Protein Sci..

[144] Patton A (2005). Endocytosis function of a ligand-gated ion channel homolog in *Caenorhabditis elegans*. Curr. Biol..

[145] Gendrel M, Rapti G, Richmond JE, Bessereau JL (2009). A secreted complement-control-related protein ensures acetylcholine receptor clustering. Nature.

[146] Boulin T (2012). Positive modulation of a Cys-loop acetylcholine receptor by an auxiliary transmembrane subunit. Nat. Neurosci..

[147] Chan JP, Hu Z, Sieburth D (2012). Recruitment of sphingosine kinase to presynaptic terminals by a conserved muscarinic signaling pathway promotes neurotransmitter release. Genes Dev..

[148] Sun L (2014). Acetylcholine promotes ROS detoxification against hypoxia/reoxygenation-induced oxidative stress through FoxO3a/PGC-1α dependent superoxide dismutase. Cell. Physiol. Biochem..

[149] Guest M (2007). The calcium-activated potassium channel, SLO-1, is required for the action of the novel cyclo-octadepsipeptide anthelmintic, emodepside *Caenorhabditis elegans*. Int. J. Parasitol..

[150] Hurd DD, Miller RM, Núñez L, Portman DS (2010). Specific α- and β-tubulin isotypes optimize the functions of sensory cilia in *Caenorhabditis elegans*. Genetics.

[151] Wang Z (2013). The EBAX-type cullin-RING E3 ligase and Hsp90 guard the protein quality of the SAX-3/Robo receptor in developing neurons. Neuron.

[152] Woo WM (2008). The *C. elegans* F-spondin family protein SPON-1 maintains cell adhesion in neural and non-neural tissues. Development.

[153] Schwarz V, Pan J, Voltmer-Irsch S, Hutter H (2009). IgCAMs redundantly control axon navigation in *Caenorhabditis elegans*. Neural Dev..

[154] Gu G, Caldwell GA, Chalfie M (1996). Genetic interactions affecting touch sensitivity in *Caenorhabditis elegans*. Proc. Natl. Acad. Sci. U. S. A..

[155] Han L (2013). Two novel DEG/ENaC channel subunits expressed in glia are needed for nose-touch sensitivity in *Caenorhabditis elegans*. J. Neurosci..

[156] Li Z (2012). Dissecting a central flip-flop circuit that integrates contradictory sensory cues in *C. elegans* feeding regulation. Nat. Commun..

[157] Tsuji N, Morales TH, Ozols VV, Carmody AB, Chandrashekar R (1999). Identification of an asparagine amidohydrolase from the filarial parasite *Dirofilaria immitis*. Int. J. Parasitol..

[158] Chen CC, Lim CY, Lee PJ, Hsu AL, Ching TT (2020). S-adenosyl methionine synthetase SAMS-5 mediates dietary restriction-induced longevity in *Caenorhabditis elegans*. PLoS ONE.

[159] Heby O (1981). Role of polyamines in the control of cell proliferation and differentiation. Differentiation.

[160] Gilad GM, Gilad VH (1991). Polyamines can protect against ischemia-induced nerve cell death in gerbil forebrain. Exp. Neurol..

[161] Ward JD (2014). Defects in the *C. elegans* acyl-CoA synthase, acs-3, and nuclear hormone receptor, nhr-25, cause sensitivity to distinct, but overlapping stresses. PLoS ONE.

[162] Wang F (2021). Saturated very long chain fatty acid configures glycosphingolipid for lysosome homeostasis in long-lived *C. elegans*. Nat. Commun..

[163] Bastiani CA, Gharib S, Simon MI, Sternberg PW (2003). *Caenorhabditis elegans* Gαq regulates egg-laying behavior via a PLCβ-independent and serotonin-dependent signaling pathway and likely functions both in the nervous system and in muscle. Genetics.

[164] Taha TA (2006). Loss of sphingosine kinase-1 activates the intrinsic pathway of programmed cell death: Modulation of sphingolipid levels and the induction of apoptosis. FASEB J..

[165] Menuz V (2009). Protection of *C. elegans* from Anoxia by HYL-2 ceramide synthase. Science.

[166] Watts JL, Ristow M (2017). Lipid and carbohydrate metabolism in *Caenorhabditis elegans*. Genetics.

[167] Teramoto T (2010). Magnesium excretion in *C. elegans* requires the activity of the GTL-2 TRPM channel. PLoS ONE.

[168] Tsunenari T (2003). Structure-function analysis of the bestrophin family of anion channels *. J. Biol. Chem..

[169] Wang Y (2013). Phylogenetic, expression, and functional analyses of anoctamin homologs in *Caenorhabditis elegans*. Am. J. Physiol..

[170] Goh KY, Inoue T (2018). A large transcribed enhancer region regulates *C. elegans* bed-3 and the development of egg laying muscles. Biochim. Biophys. Acta..

[171] Currie E (2007). Role of the *Caenorhabditis elegans* multidrug resistance Gene, mrp-4, in gut granule differentiation. . Genetics.

[172] Schroeder LK (2007). Function of the *Caenorhabditis elegans* ABC transporter PGP-2 in the biogenesis of a lysosome-related fat storage organelle. Mol. Biol. Cell.

[173] Kage-Nakadai E, Uehara T, Mitani S (2011). H+/myo-inositol transporter genes, hmit-1.1 and hmit-1.2, have roles in the osmoprotective response in *Caenorhabditis elegans*. Biochem. Biophys. Res. Commun..

[174] Pao SS, Paulsen IT, Saier MH (1998). Major facilitator superfamily. Microbiol. Mol. Biol. Rev..

[175] Romanelli-Credrez L, Doitsidou M, Alkema MJ, Salinas G (2020). HIF-1 has a central role in *Caenorhabditis elegans* organismal response to selenium. Front. Genet..

[176] Filipovic MR, Zivanovic J, Alvarez B, Banerjee R (2018). Chemical biology of H2S signaling through persulfidation. Chem. Rev..

[177] Diaz-Vivancos P, De Simone A, Kiddle G, Foyer CH (2015). Glutathione: Linking cell proliferation to oxidative stress. Free Radic. Biol. Med..

[178] Morimoto-Tomita M, Uchimura K, Werb Z, Hemmerich S, Rosen SD (2002). Cloning and characterization of two extracellular heparin-degrading endosulfatases in mice and humans *. J. Biol. Chem..

[179] Rose P, Moore PK, Zhu YZ (2016). H2S biosynthesis and catabolism: New insights from molecular studies. Cell. Mol. Life Sci..

[180] Qabazard B (2014). Hydrogen sulfide is an endogenous regulator of aging in *Caenorhabditis elegans*. Antioxid. Redox Signal..

[181] Cavanaugh CM, McKiness ZP, Newton ILG, Stewart FJ (2013). Marine chemosynthetic symbioses. Prokaryotes Prokaryotic Biol. Symbiotic Assoc..

[182] Grabherr MG (2011). Full-length transcriptome assembly from RNA-Seq data without a reference genome. Nat. Biotechnol..

[183] Cerveau N, Jackson DJ (2016). Combining independent de novo assemblies optimizes the coding transcriptome for nonconventional model eukaryotic organisms. BMC Bioinform..

[184] Fu L, Niu B, Zhu Z, Wu S, Li W (2012). CD-HIT: Accelerated for clustering the next-generation sequencing data. Bioinformatics.

[185] Huson DH, Auch AF, Qi J, Schuster SC (2007). MEGAN analysis of metagenomic data. Genome Res..

[186] Bryant DM (2017). A tissue-mapped axolotl De Novo transcriptome enables identification of limb regeneration factors. Cell Rep..

[187] Li B, Dewey CN (2011). RSEM: Accurate transcript quantification from RNA-Seq data with or without a reference genome. BMC Bioinform..

[188] Robinson MD, McCarthy DJ, Smyth GK (2009). edgeR: A Bioconductor package for differential expression analysis of digital gene expression data. Bioinformatics.

[189] R Core Team (2020). R: A Language and Environment for Statistical Computing.

[190] Rapaport F (2013). Comprehensive evaluation of differential gene expression analysis methods for RNA-seq data. Genome Biol..

[191] Cantalapiedra CP, Hernandez-Plaza A, Letunic I, Bork P, Huerta-Cepas J (2021). eggNOG-mapper v2: Functional annotation, orthology assignments, and domain prediction at the metagenomic scale. Mol. Biol. Evol..

[192] Huerta-Cepas J (2019). eggNOG 5.0: A hierarchical, functionally and phylogenetically annotated orthology resource based on 5090 organisms and 2502 viruses. Nucleic Acids Res..

[193] Buchfink B, Reuter K, Drost HG (2021). Sensitive protein alignments at tree-of-life scale using DIAMOND. Nat. Methods.

[194] Altschul SF, Gish W, Miller W, Myers EW, Lipman DJ (1990). Basic local alignment search tool. J. Mol. Biol..

[195] Harris TW (2020). WormBase: A modern model organism information resource. Nucleic Acids Res..

