# Supplementary figures and images for: Differential regulation of degradation and immune pathways underlies adaptation of the ectosymbiotic nematode Laxus oneistus to oxic-anoxic interfaces
Source: Sci Rep. 2022 Jun 13;12:9725. doi: 10.1038/s41598-022-13235-9 (PMC9192688; doi:10.1038/s41598-022-13235-9)

**A**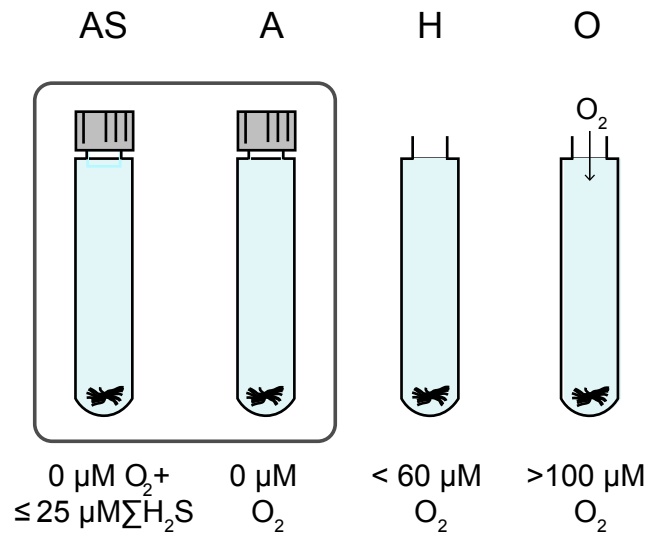**B**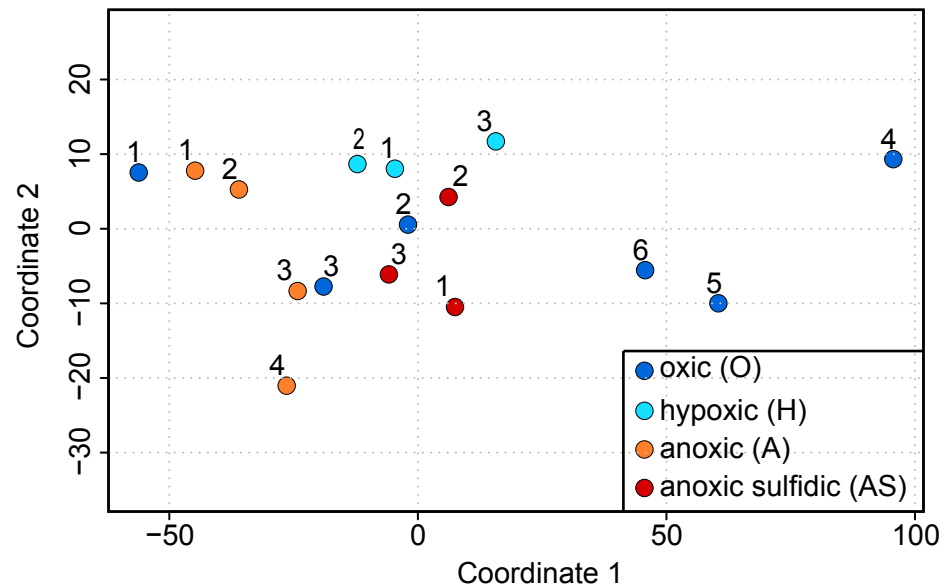**C**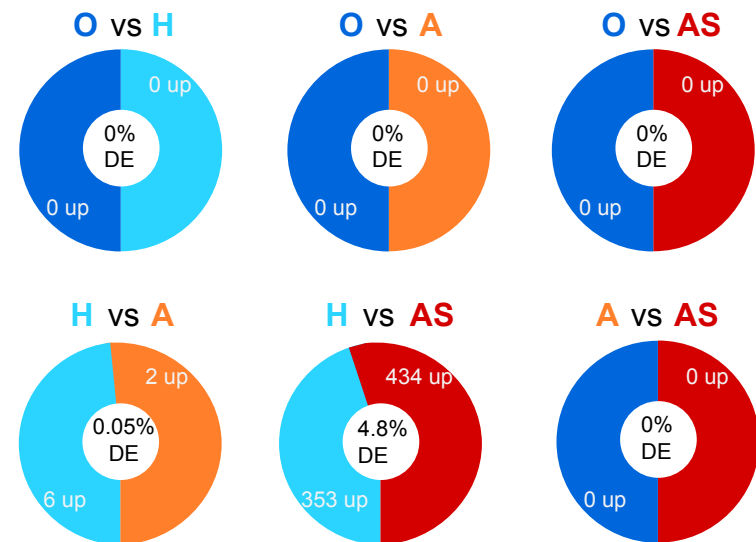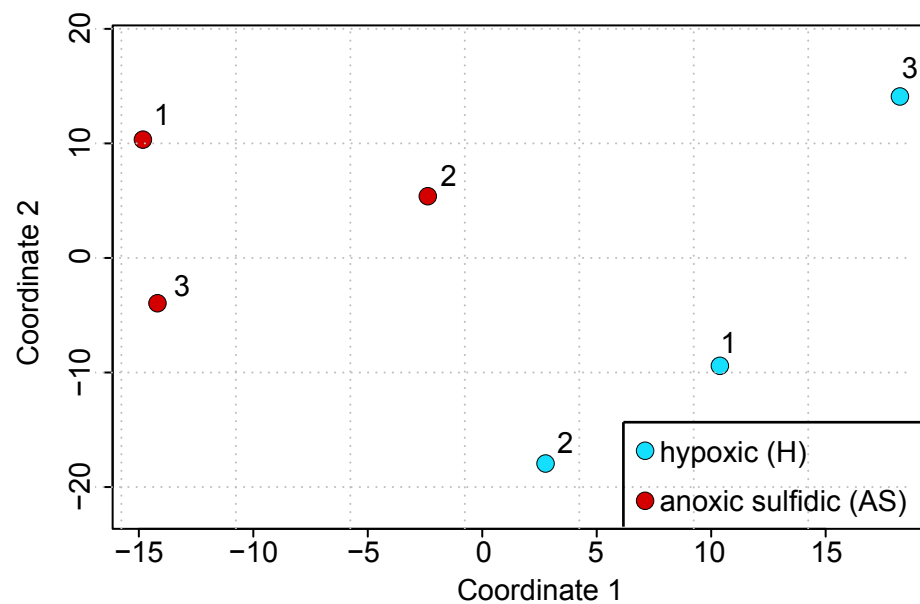

Supplement: Supplementary file 3 — Supplementary Figure S1. [file 41598_2022_13235_MOESM3_ESM.pdf]

A

0 up

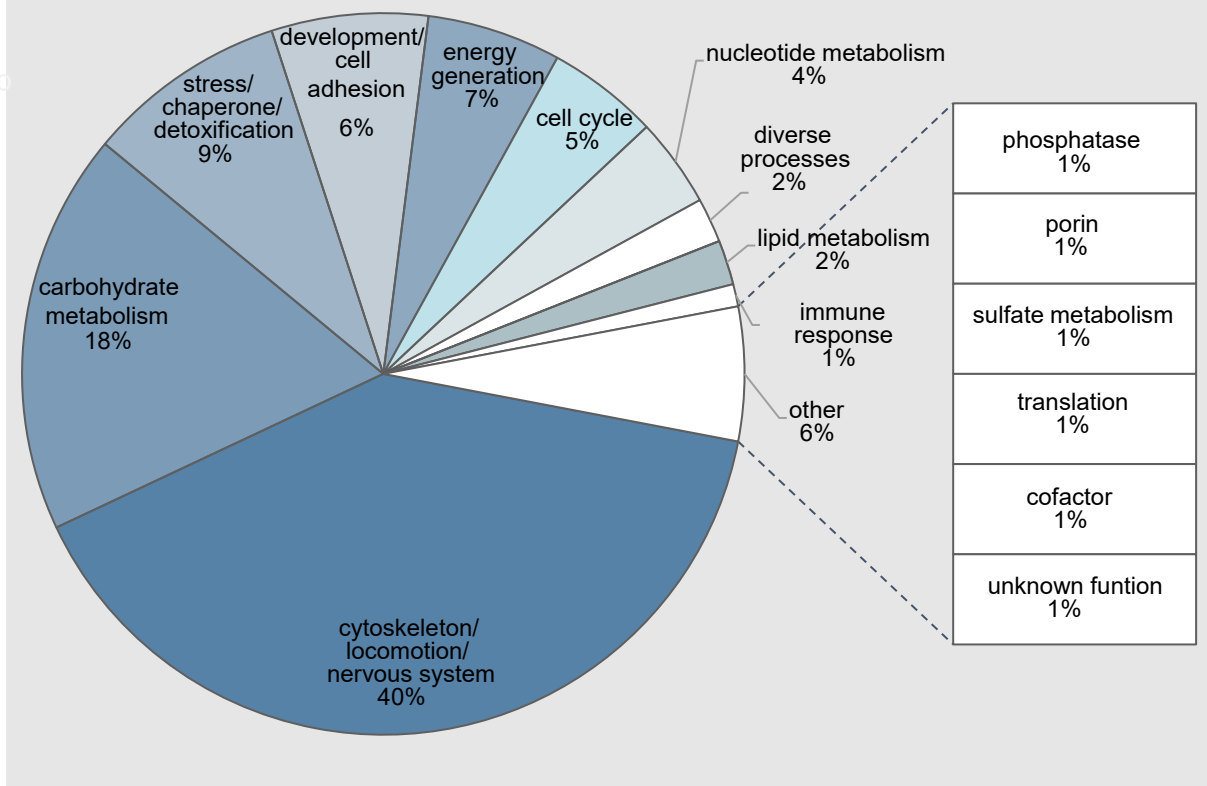

B

Log2TPM

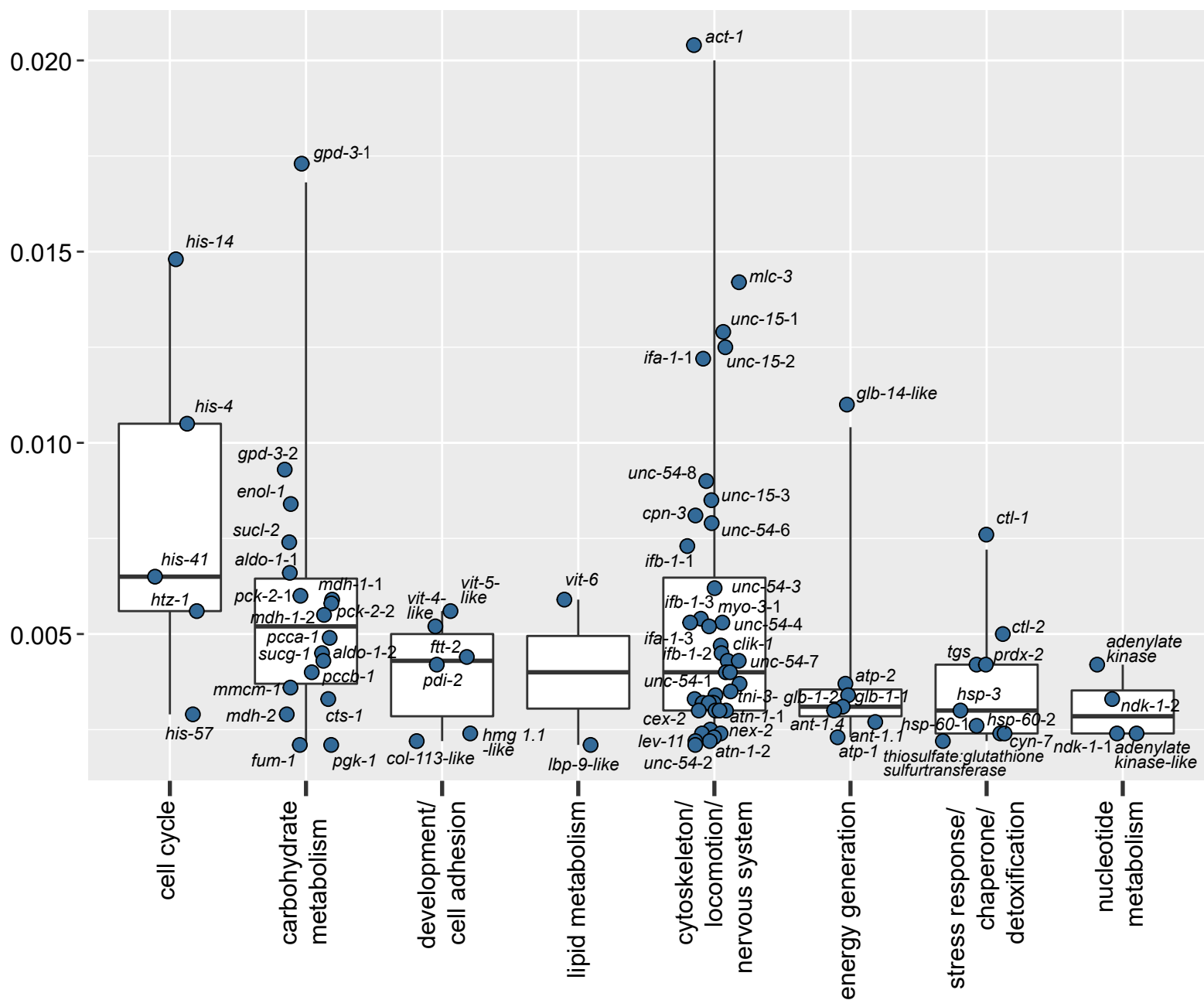

Supplement: Supplementary file 4 — Supplementary Figure S2. [file 41598_2022_13235_MOESM4_ESM.pdf]

A

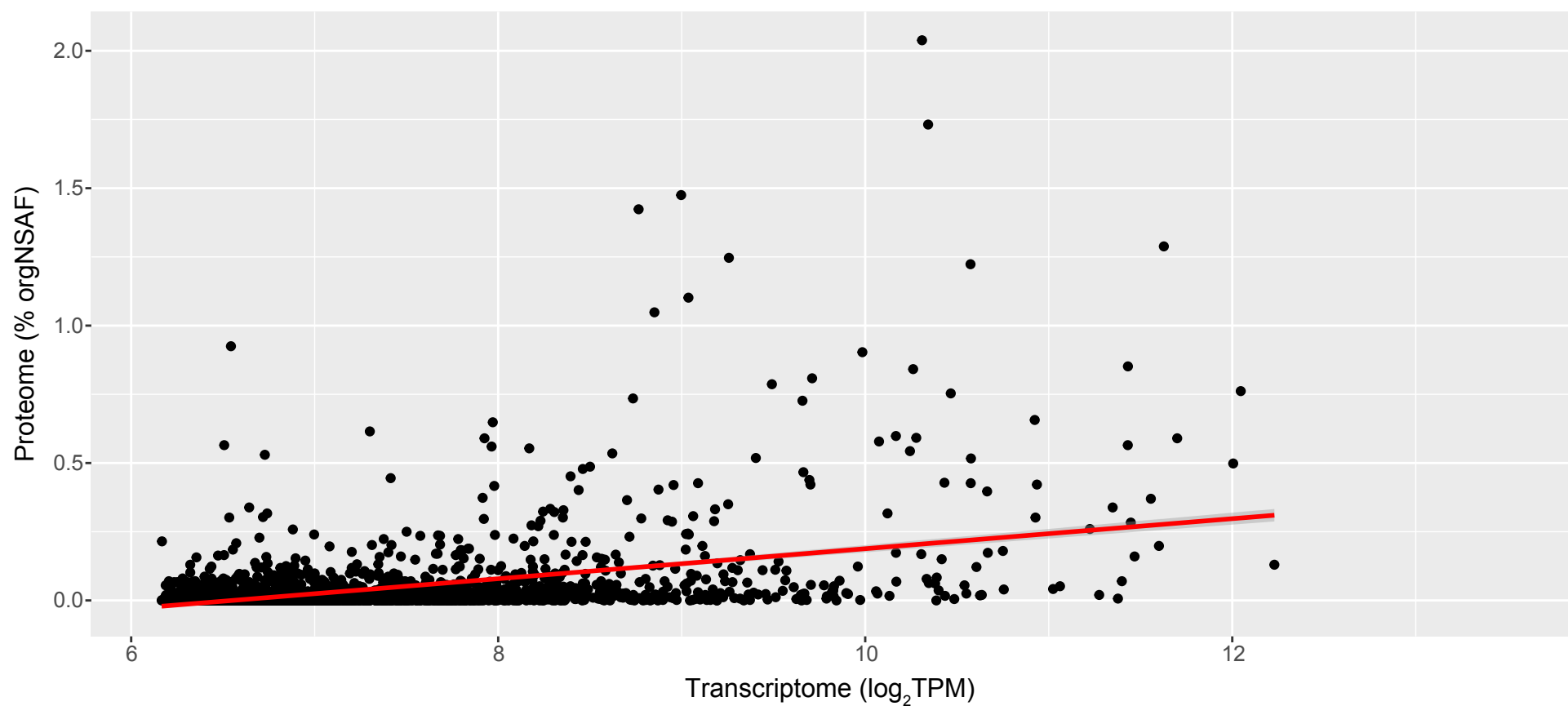

B

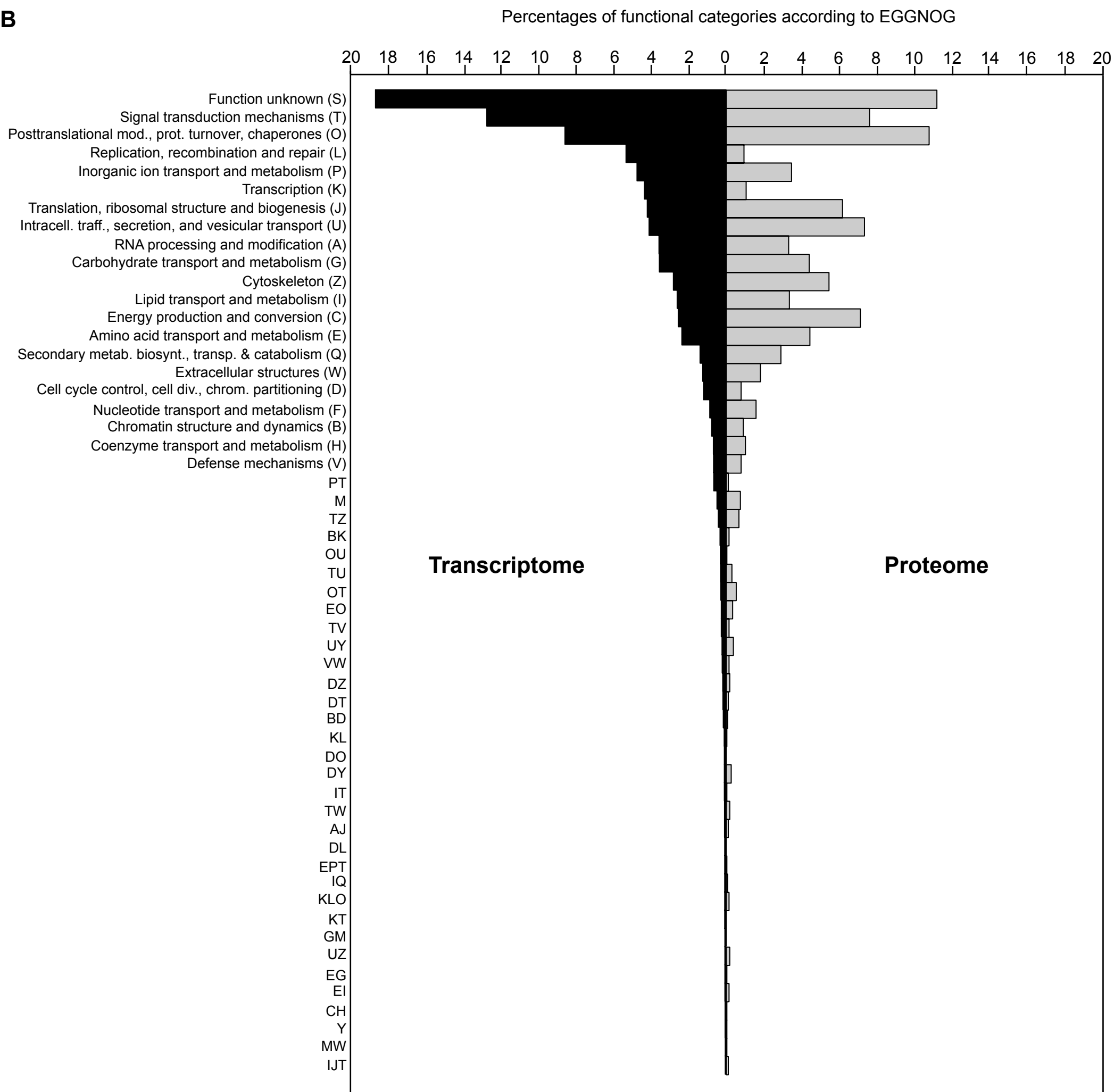

Supplement: Supplementary file 5 — Supplementary Figure S3. [file 41598_2022_13235_MOESM5_ESM.pdf]

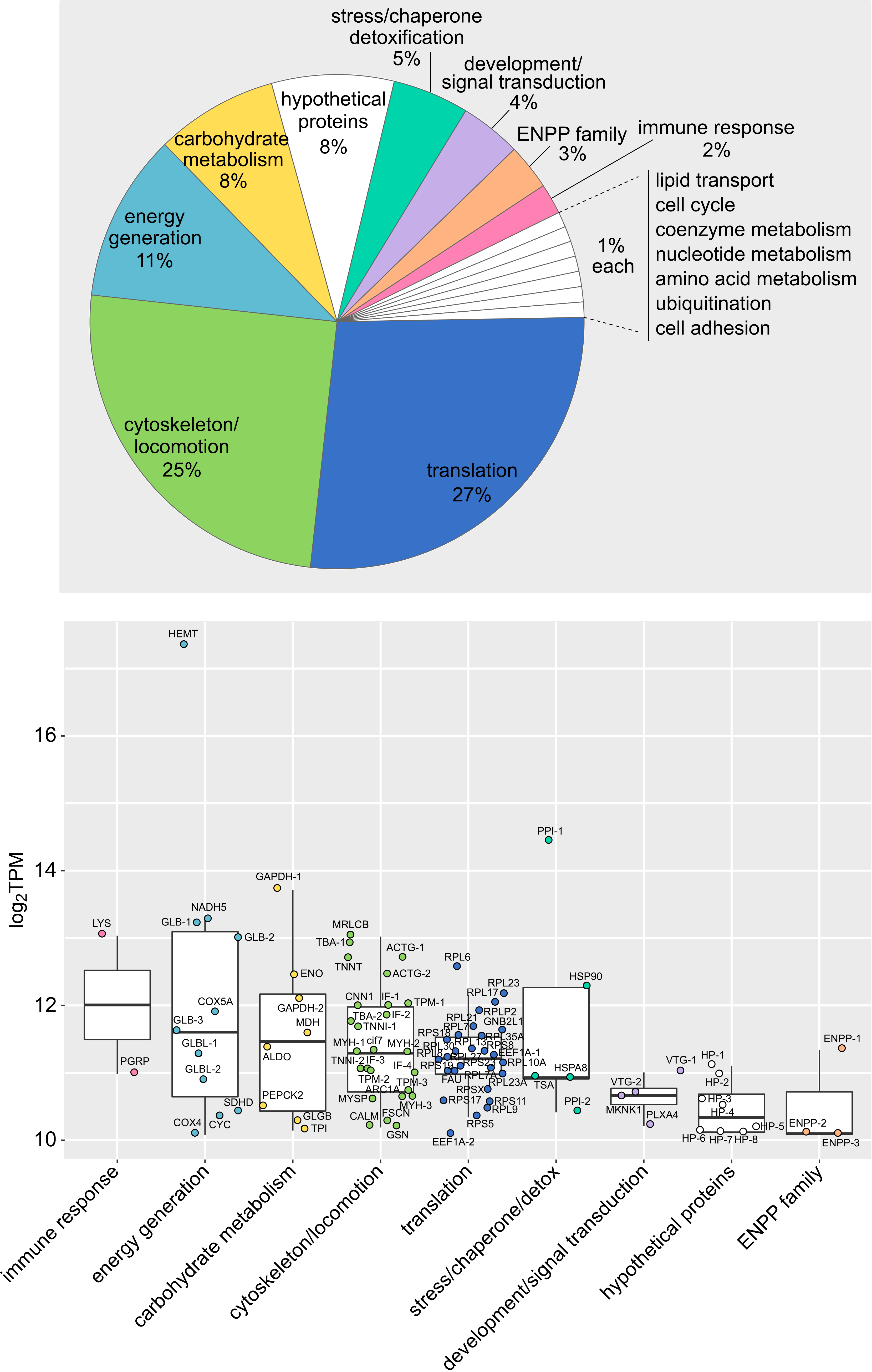

Supplement: Supplementary file 6 — Supplementary Figure S4. [file 41598_2022_13235_MOESM6_ESM.png]

anoxic (A)

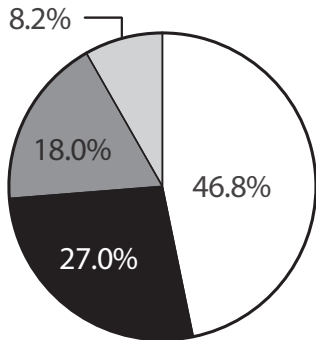

oxic (O)

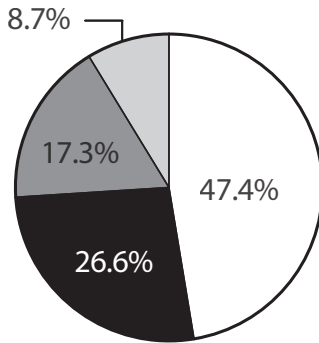

- Phospholipids
- Glycerolipids
- Sphingolipids
- Unidentified

Supplement: Supplementary file 7 — Supplementary Figure S5. [file 41598_2022_13235_MOESM7_ESM.pdf]
