# Supplementary material for: Differential regulation of degradation and immune pathways underlies adaptation of the ectosymbiotic nematode Laxus oneistus to oxic-anoxic interfaces
Source: Sci Rep. 2022 Jun 13;12:9725. doi: 10.1038/s41598-022-13235-9 (PMC9192688; doi:10.1038/s41598-022-13235-9)
Supplement: Supplementary file 13 — Supplementary Legends. [file 41598_2022_13235_MOESM13_ESM.docx]

**SUPPLEMENTARY FIGURE, VIDEO AND MATERIAL LEGENDS**

**Figure S1. Experimental conditions, sample similarity and differential expression.** (A) Experimental setup was previously described (Paredes et al. 2021). Briefly, nematodes were subjected for 24 h to the following conditions: anoxic with sulfide (0 mM O_2_ and 25 mM sodium sulfide added at T0; AS), anoxic without sulfide (0 mM O_2_ A), hypoxic (<60 mM O_2_ at the end of the incubation, H), and oxic (>100mM O_2_ at end of the incubation; O). The box around the anoxic incubation vials illustrates that these incubations were carried out in a polyethylene glove bag. (B) Similarity between transcriptome samples based on Euclidean distances between expression values (log_2_TPM), and visualized by means of multidimensional scaling. (C) Differential gene expression (DE) analysis between incubations. Genes were considered differentially expressed if their expression changed 1.5-fold with a false-discovery rate (FDR) of ≤ 0.05. Maximal differential expression of all expressed genes (4.8%) was between the H and the AS condition.

**Figure S2. Statistical analysis, relative transcript abundance and expression levels of the top 100 detected proteins of *L. oneistus* across all conditions.** (A) Relative protein abundance (%) of the top 100 detected proteins present in a particular manually curated functional category. The top 100 proteins were collected by averaging the expression values across all replicates of all incubations (Figure S1A, Data S2). Functional classifications were extracted from UniProt and from comprehensive literature search focused mainly on *C. elegans,* and confirmed with the automatically annotated eggNOG classification (Data S1). (B) Median gene expression levels of selected *L. oneistus* manually annotated functional categories of the top 100 expressed proteins. Each dot represents the average %OrgNSAF per protein across all replicates of all incubations. Note that some categories were created with genes of overlapping functions (e.g., cytoskeleton/locomotion/nervous system). All protein names (or locus tags for unidentified protein names) are listed in Data S2.

**Figure S3. Transcriptomics versus proteomics comparison.** (A) Scatter plot of per-gene averaged log_2_TPM and orgNSAF% values (Data S1) of the oxic and anoxic incubations for the transcriptome (x axis) and proteome (y axis), respectively. Spearman correlation between transcriptome and proteome is displayed as a red line (ρ = 0.4, *p*-value < 0.01). (B) Automatic classification of all identified transcripts and proteins based on their functional EggNOG category.

**Figure S4.** **Relative transcript abundance and expression levels of the top 100 expressed genes of *O. algarvensis* across all conditions.** (A) Relative transcript abundance (%) of the top 100 expressed genes with a manually curated functional category. The top 100 expressed genes were collected by averaging the expression values (log_2_TPM) across all replicates of all incubations (for details on the incubations see Paredes et al., 2021). Functional classifications were extracted from UniProt and from comprehensive literature search focused mainly on *C. elegans*). (B) Median gene expression levels of selected *O*. *algarvensis* manually annotated functional categories of the top 100 expressed genes. Metabolic processes include both differentially and constitutively expressed genes. Each dot represents the average log_2_TPM value per gene across all replicates of all incubations.

**Figure S5. *L. oneistus* lipid composition in anoxic and oxic conditions after 24 h.** Major lipid classes and their abundance relative to all lipids detected. For details on methodology see Supplementary Information 3.

**Table S1.** Metabolites detected in at least two biological replicates of either the holobiont fraction (*Laxus oneistus* and its ectosymbiont) or in the symbiont fraction (see Supplementary Information 3). RT: retention time. Area: area of a peak from a specific compound detected in the GC-MS chromatograms. Grey boxes: no metabolites detected. Blank boxes: unknown metabolites that are either below the detected threshold (< 700) or might be products of derivatization reagents. Cholestane and ribitol were used as internal standards.

**Data S1.** *Laxus oneistus* genes, functional annotations, transcript and protein expression.

**Data S2**. *Laxus oneistus* top 100 expressed genes (based on RNA-Seq) and proteins (based on proteomics).

**Supplemental video 1.** A batch of 50 *Laxus oneistus* after 6 days in anoxic seawater.

**Supplemental video 2.** A batch of 50 *Laxus oneistus* at the beginning (T0) of the incubations.

**Supplemental video 3.** A batch of 50 *Laxus oneistus* after 1 day (T24 h) in anoxic sulfidic seawater (0 % air saturation, 25 µM H_2_S).

**Supplemental video 4.** A batch of 50 *Laxus oneistus* after 1 day (T24 h) in oxic seawater (87 % air saturation, 0 µM H_2_S).
